# Factors governing attachment of *Rhizobium leguminosarum* to legume roots at acid, neutral, and alkaline pHs

**DOI:** 10.1128/msystems.00422-24

**Published:** 2024-08-21

**Authors:** Jack D. Parsons, Clare R. Cocker, Alison K. East, Rachel M. Wheatley, Vinoy K. Ramachandran, Farnusch Kaschani, Markus Kaiser, Philip S. Poole

**Affiliations:** 1 Department of Biology, University of Oxford, Oxford, United Kingdom; 2 Department of Chemical Biology, ZMB, University Duisburg-Essen, Essen, Germany; 3 Analytics Core Facility Essen (ACE), University Duisburg-Essen, ZMB, Essen, Germany; University of Pretoria, Hatfield, South Africa

**Keywords:** root, attachment, rhizobium, legume, pea, adhesin

## Abstract

Rhizobial attachment to host legume roots is the first physical interaction of bacteria and plants in symbiotic nitrogen fixation. The pH-dependent primary attachment of *Rhizobium leguminosarum* biovar viciae 3841 to *Pisum sativum* (pea) roots was investigated by genome-wide insertion sequencing, luminescence-based attachment assays, and proteomic analysis. Under acid, neutral, or alkaline pH, a total of 115 genes are needed for primary attachment under one or more environmental pH, with 22 genes required for all. These include components of cell surfaces and membranes, together with enzymes that construct and modify them. Mechanisms of dealing with stress also play a part; however, exact requirements vary depending on environmental pH. RNASeq showed that knocking out the two transcriptional regulators required for attachment causes massive changes in the bacterial cell surface. Approximately half of the 54 proteins required for attachment at pH 7.0 have a role in the later stages of nodule formation. We found no evidence for a single rhicadhesin responsible for alkaline attachment, although sonicated cell surface fractions inhibited root attachment at alkaline pH. Our results demonstrate the complexity of primary root attachment and illustrate the diversity of mechanisms involved.

**IMPORTANCE:**

The first step by which bacteria interact with plant roots is by attachment. In this study, we use a combination of insertion sequencing and biochemical analysis to determine how bacteria attach to pea roots and how this is influenced by pH. We identify several key adhesins, which are molecules that enable bacteria to stick to roots. This includes a novel filamentous hemagglutinin which is needed at all pHs for attachment. Overall, 115 proteins are required for attachment at one or more pHs.

## INTRODUCTION

Rhizobia are diazotrophic soil Alphaproteobacteria that form nitrogen-fixing symbioses with host legume plants. Formation of these symbioses between rhizobia and their host legumes requires a complex and specific process of molecular cross-talk beginning in the rhizosphere (for a full review, see references 1, 2). In the initial stages of symbiosis, host plants secrete a range of messenger molecules [including (iso)flavonoids] which induce rhizobial synthesis of Nodulation (Nod) factor (a decorated lipochitooligosaccharide), a specificity signal that has a role in triggering infection of the host plant and nodule organogenesis by root hair curling engulfing bacteria (1). In the very first physical interaction, prior to root hair curling, rhizobia attach to the roots of the host legume. This attachment occurs in two distinct phases: primary attachment, defined as early-stage reversible interactions of single bacterial cells, and secondary attachment, defined as later-stage irreversible binding, often mediated by extracellular fibrils, and dependent on successful primary attachment (3). Primary attachment of rhizobia to legume roots is thought necessary for the subsequent successful formation of nitrogen-fixing symbioses, especially under the competitive conditions that exist in soil (4).

Much of the existing literature concerning primary root attachment in *Rhizobium*-legume symbioses, prior to biofilm formation and colonization of the root surface, describes a pH-dependent bacterial system based on a glucomannan/rhicadhesin duality (reviewed in references 3, 5). In this model, under mildly acidic soil conditions, rhizobia use polarly located glucomannan to bind to plant-root lectins (i.e., any plant protein with at least one non-catalytic domain showing specific and reversible carbohydrate-binding affinity) (4, 6), while in mildly alkaline soil where lectins disassociate from roots, rhizobia make use of rhicadhesin (a putative 14 kDa calcium-binding protein) (7) to bind a (as yet uncharacterized) plant-root receptor for primary attachment (3, 5). Despite the reported purification of the rhicadhesin protein many years ago (7), the gene has never been conclusively identified. It is assumed that there is some overlap of these two systems under neutral soil conditions. However, the glucomannan/rhicadhesin hypothesis is likely to be incomplete as a model for primary attachment. First, neither the rhicadhesin gene nor its plant receptor has been identified. Second, while other factors are important in primary attachment, they are often not taken into account. These include van der Waals forces, electrostatic, and hydrophobic interactions (3, 8, 9), as well as a range of molecular bacterial factors. Mutation of the extracellular polysaccharide (EPS) biosynthesis gene *pssA* in *Rhizobium leguminosarum* biovar viciae disrupted the ability to attach and form biofilms on inert surfaces, although it was not tested in root attachment assays (4, 8). Attempts to clone the rhicadhesin gene using an approach based on phage display isolated several *Rhizobium*-adhering proteins (Raps) able to agglutinate cells, as well as promote biofilm formation and short-term attachment (4 h) to legume roots (10–12). Furthermore, mutation of the regulator *praR* in *R. leguminosarum* bv. viciae was shown to enhance biofilm formation both on inert surfaces and legume roots (2-h assay), primarily through upregulation of Rap expression (13).

Therefore, although the glucomannan-rhicadhesin model is often used to explain primary root attachment in *Rhizobium*-legume symbioses, multiple other factors are likely to play a role. The rhizosphere is a complex and highly heterogeneous environment where both biotic and abiotic conditions vary (14–16). With existing literature linking diverse proteins and processes to attachment (4, 8, 10–13), it suggests that the suite of factors necessary for primary attachment to plant roots may be more complex than previously thought.

Existing tools for the investigation of attachment, while providing useful approaches for characterizing novel factors, are proving to be rate-limiting in fully characterizing primary attachment, in part, due to the wide range of different approaches. Primary attachment assays, ranging from 1 to 4 h in duration, have used wild-type (WT) or mutant rhizobia and phase contrast microscopy to count bacteria attached to roots, or vortexing/sonication of roots to remove bacteria before plating them on growth media for colony enumeration (12, 17–19). However, these methods are low-throughput, may suffer from “region bias” when counting attached bacteria using microscopy, and result in loss of spatial attachment data if bacteria are removed before counting. Frederix et al. (13) developed a scintillation-based attachment assay using radiolabeled rhizobia and, subsequently, a luminescence (Lux)-based attachment assay by making use of rhizobia carrying a stably inherited plasmid encoding the *luxCDABE* operon. Each of the above methods made use of excised root sections, introducing an unnatural source of root exudation from the wound site, potentially affecting attachment. Furthermore, none were tested for their suitability in conducting primary attachment assays across a range of pHs. Initial reports of the effects of pH on rhizobial root attachment come from a variety of different methods with some growing the bacteria at one pH and assessing attachment at another (4, 6).

In this study, we have developed and standardized a 1-h primary attachment assay to determine genetic factors important for primary root attachment of *Rhizobium leguminosarum* biovar viciae 3841 (Rlv3841) (20) to roots of the host legume *Pisum sativum* (pea). In contrast to studies of bacterial colonization of plant roots (e.g., references 21, 22), there have been no reports of genome-wide experiments examining primary attachment. Using *mariner* transposon-based insertion sequencing (INSeq) (23), attachment experiments were performed under acid, neutral, and alkaline plant growth conditions. INSeq enables a holistic study of gene fitness under conditions of interest using high-throughput sequencing of a bacterial insertion mutant library isolated from different environmental conditions (in this case from pea roots at acid, neutral, and alkaline pH), combined with hidden Markov model (HMM) analysis (24–26). INSeq results were compared with data from whole-root Lux-based bacterial attachment assays performed under the same three pH conditions. We identified key genes for primary attachment under all conditions tested (pH 6.5, pH 7.0, and pH 7.5), as well as at specific pHs. By combining INSeq, Lux-based attachment data, and a proteomics approach, we attempted to identify the rhicadhesin gene.

## MATERIALS AND METHODS

### Strains, plasmids, and culture conditions

All strains and plasmids used are listed in Table S1. Rhizobial strains were grown at 28°C on universal minimal salts (UMS) medium (25) supplemented with 30 mM sodium pyruvate, and 10 mM ammonium chloride, and adjusted to pH 6.5, pH 7.0, or pH 7.5. For solid media, 1% (wt/vol) agar was added. All *Escherichia coli* strains were grown at 37°C in Lennox (L)-broth or on L-agar (27). Antibiotics were used at the following concentrations (μg mL^−1^), unless otherwise stated: neomycin, 80; streptomycin, 500; and tetracycline, 5. The donor *E. coli* strain SM10*λpir* [carrying the *mariner* transposon INSeq pSAM_Rl vector (23)] was supplemented with 50 µg mL^−1^ neomycin to select for the plasmid and 100 µg mL^−1^ ampicillin to select for *E. coli*. Following the recovery of attached bacteria in the INSeq attachment assays, bacteria were regrown for 12 h in tryptone-yeast extract (TY) (25), prior to DNA extraction.

To assess the ability of bacteria lacking a specific gene in primary attachment to roots, *Rhizobium* mutant strains were made using single-crossover integration pK19mob-mutagenesis which uses homologous recombination to disrupt the gene in the bacterial genome. An internal fragment of the gene was first cloned in vector pK19 using PCR amplification of the gene fragment from Rlv3841 template DNA (28). Following the transformation of Rlv3841 and the selection of neomycin-resistant colonies, integration of pK19 into the expected gene was confirmed by PCR mapping (primers listed in Table S2). For all Lux-based attachment assays (13), *Rhizobium* strains were labeled by introduction of pIJ11282, a plasmid with *luxCDABE* constitutively expressed from *nptII* promoter and stably maintained in rhizobia (13). Plasmid pIJ11282 was introduced into rhizobial strains according to the method of Figurski and Helinski (29).

### Buffering capacity of vermiculite

To assess the ability of vermiculite to buffer pH changes, rooting solution (25 mL) (consisting of 1 mM CaCl_2_·2H_2_O, 100 µM KCl, 800 µM MgSO_4_·7H_2_O, 10 µM Fe EDTA, 35 µM H_3_BO_3_, 9 µM MnCl_2_·4H_2_O, 0.8 µM ZnCl_2_, 0.5 µM Na_2_MoO_4_·2H_2_O, 0.3 µM CuSO_4_·5H_2_O, 4 mM KH_2_PO_4_, and 4 mM Na_2_HPO_4_) was adjusted to pH 6.5, 7.0, or 7.5 using hydrochloric acid or sodium hydroxide solution and added to 2.5 g fine vermiculite (Sinclair) in 50 mL Falcon tubes, in triplicate. Tubes were rotated gently at 20 rpm and the pH of the rooting solution was measured using a pH meter (Hanna) at time intervals of up to 72 h. Although slight pH increases were seen over time, the pH never increased by more than 0.16 above the initial pH (Fig. S1), which was regarded as acceptable for the purposes of these experiments.

### Plant growth and root attachment assays

Plants were grown as follows: seeds of *Pisum sativum* (var. Avola) were sterilized in a 5% sodium hypochlorite solution for 5 min before washing six times with distilled water. Washed fine vermiculite (30 g) was placed in 100 mL boiling tubes, together with 25 mL of rooting solution (adjusted to pH 6.5, pH 7.0, or pH 7.5, depending on the experiment). Boiling tubes were sterilized, and pea seeds planted under sterile conditions were grown for 7 days. Three days prior to the experiment, Rlv3841-based strains (*mariner* transposon library or individual Lux-labeled strains) were streaked on UMS agar slopes. For INSeq experiments, all slopes were at pH 7.0. For Lux-based attachment assays, the pH of the slopes was adjusted to the test pH using hydrochloric acid or sodium hydroxide solution. For both INSeq experiments and Lux-based assays, the same procedure to assess attachment was followed: bacteria were resuspended in 15 mM MES/HEPES (pH adjusted as appropriate) to an OD_600_ of 0.1. This starting inoculum density was used to inoculate plants, *n* = 10 (approximately 2 × 10^6^ CFU per root [Fig. S2]). Plants were removed from vermiculite, washed in 15 mM MES/HEPES (of the correct pH), and their roots submerged in 50 mL of the bacterial suspension for 1 h, with gentle (20 rpm) agitation to ensure the solution was well-mixed and oxygenated. After 1 h, roots were removed and washed by dipping in 15 mM MES/HEPES (of the correct pH). For INSeq experiments, roots were excised from the seed and vortexed (Heidolph Multi Reax) at maximum speed for 10 min in 50-ml Falcon tubes with 20 mL 15 mM MES/HEPES. Roots were discarded, and the pooled liquid was filtered through three layers of sterile muslin cloth before re-growing bacteria in 50 mL TY media at 28°C for 12 h (with shaking at 180 rpm). This step was included to boost bacterial DNA, helping to minimize contamination by plant material. DNA was then isolated using a Qiagen DNeasy Blood and Tissue Kit for Gram-negative bacteria according to the manufacturer’s protocol with the modifications described in reference 25. For the input library, DNA was isolated directly from 50 mL of OD_600_ = 0.1 resuspension (i.e., the same bacterial suspension, prior to pH adjustment, as the one into roots was dipped) as described above. INSeq was performed in triplicate for each test condition (root attachment at acid, neutral, and alkaline) and the three input libraries (see Fig. S3).

For Lux-based bacterial attachment assays (13), weighed pea roots (*n* = 10) were imaged using a NightOWL LB 983 imaging system (Berthold). Root-attached luminescence was normalized using inoculum luminescence and measured in triplicate using a GloMax plate reader (Promega). Bacterial attachment to roots is expressed as relative light units (RLU)/g of root (13). To assess the effect of a crude preparation of adhesin, sterilized peas were germinated for 5 days on water agar. Roots were excised from seed and seedlings, and preincubated with the crude adhesin fraction (600 µg protein) for 1 h before washing by dipping in 15 mM MES/HEPES before bacterial attachment was assessed in Lux-based assays.

### *mariner* transposon library construction

To assess rhizobial genes involved in primary attachment to pea roots at acid, neutral, and alkaline pH, a *mariner* transposon library was constructed as previously described by Wheatley et al. (25). Briefly, donor *E. coli* was grown in L-broth overnight and Rlv3841 was grown on a TY agar slope. Cultures were pelleted, resuspended in TY, and pooled. Cells were further pelleted and resuspended in TY before spotting cell suspensions on nitrocellulose filters on TY agar plates and incubated at 28°C overnight. Filters were resuspended in UMS with 15% glycerol before the enumeration of transposon insertions in Rlv3841 on TY agar supplemented with streptomycin and neomycin and pooling of mutants.

### Library preparation and sequencing

Following DNA isolation, transposon tags were prepared for DNA sequencing as described in reference 25. Briefly, linear PCR products were amplified using BioSAM primers (23) with 1,000 ng DNA (see Table S2 for primer and adaptor sequences). Biotinylated linear PCR products were bound to Pierce streptavidin magnetic beads (Thermo Scientific) and enzymatic library preparation was performed. A custom INSeq library adaptor was used for adaptor ligation and final PCR amplification was performed using one of 12 different barcoding primers (Table S2). The final sequencing template for each group (187 bp in length) was purified using SizeSelect II agarose gels (Invitrogen) and analyzed using a Bioanalyzer High Sensitivity DNA chip (Agilent Technologies). Libraries were diluted to 100 pM and pooled. Libraries were subjected to automatic template preparation using an Ion Chef (Life Technologies) and DNA sequencing was performed on an Ion Proton Sequencer (ThermoFisher Scientific).

### Transposon insertion analysis and HMM

Sequencing reads were analyzed on a Linux server using a custom-written Perl script as previously described (23, 25) with Cutadapt v 1.16 (30) (used for quality trimming and removal of adapter sequences, and the resulting 15- to 16-bp transposon-tags were checked for a leading TA motif with a custom-written Perl script), the Bowtie short read aligner v 1.3.1 (31) (to map the tags to the reference genome Rlv3841), and the files converted to .wig format by a custom-written Perl script (23, 25). The Tn-HMM Python module v 1.0.3 (32) was used to calculate the HMM state of each TA site and then determine the state of all TA sites within gene boundaries to assign each gene a state as a whole. Finally, it was used to quantify the number of genes assigned to each state. HMM analysis assigned genes to one of four classification states: essential (ES), growth deficient (GD), neutral (NE), and growth advantaged (GA) (32). However, for attachment experiments, the following were adopted: essential (ES), defective (DE), neutral (NE), or advantaged (AD), as used by Wheatley et al. (26). HMM categories were averaged (by selecting the mode) across the three biological replicates per test condition. Manual curation of those genes lacking a consensus classification was performed. This was achieved by visualizing INSEQ reads mapped to the Rlv3841 genome using the Integrative Genomics Viewer v 2.4.0 platform and assessing TA insertion profiles between input and experimental samples. Visualization enabled direct comparison of numbers of TA site insertions mapping in and around gene loci between input and experimental samples. This provided further control for assessing fitness impacts under input and experimental conditions, enabling an indicative consensus state call to be assigned. Genes are listed in Data sheets S1 and S2 (marked mc [manually curated]).

### Crude adhesin isolation and proteomics

To examine bacterial cell surface adhesins, Rlv3841 was grown overnight at 28°C in 250 mL UMS (pH 7.5) with shaking at 180 rpm. Cell fractionation and isolation of the crude adhesin fraction were carried out as previously described (33). Briefly, cells were harvested by centrifugation at OD_600_ = 0.7, and the cell pellet was washed and resuspended in 25 mM phosphate buffer pH 7.5. Cells were sheared using a 705 Sonic Dismembrator (Fisher Scientific) at an amplitude of 5. The suspension was centrifuged at 12,000 × *g* for 10 min at 4°C, and the resulting supernatant was then centrifuged at 100,000 × *g* (Beckmann TL-100 ultracentrifuge) for 2 h at 4°C. The supernatant obtained at this stage formed the crude adhesin fraction. Prior to root attachment assays, the concentration of protein in the crude adhesin fraction was determined using a Pierce bicinchoninic acid (BCA) assay (Thermo Scientific) according to the manufacturer’s instructions. Root attachment assays were performed following pre-incubation for 1 h with 600 µg crude adhesin protein. Separation of crude adhesin was achieved using Nu-PAGE Bis-Tris gels (Thermo Scientific) according to the manufacturer’s instructions. For protein visualization, SYPRO Ruby staining (Sigma Aldrich) was used according to the manufacturer’s “rapid” protocol. Imaging was carried out using a BioRad ChemiDoc XRS + imaging system with ImageLab (BioRad). For proteomic analysis, bands of interest were excised and subjected to liquid chromatography-mass spectrometry (LC-MS). Calcium-binding potential of candidate peptides was assessed through the IonCom server tool (34).

### In-gel digestion with trypsin

To purify the proteins, bands of interest were cut out of the SDS-PAGE gel and subjected to in-gel digestion (IGD following a published protocol (35). The acidified tryptic digests were finally desalted on homemade 2-disc C18 StageTips as described (36). After elution from the StageTips, samples were dried using a vacuum concentrator (Eppendorf), and the peptides were taken up in 10 µL 0.1% formic acid solution.

### LC-MS/MS settings

Experiments were performed on an Orbitrap Elite instrument (Thermo) (37) that was coupled to an EASY-nLC 1000 liquid chromatography (LC) system (Thermo). The LC was operated in the one-column mode. The analytical column was a fused silica capillary (75 µm × 35 cm) with an integrated PicoFrit emitter (New Objective) packed in-house with Reprosil-Pur 120 C18-AQ 1.9 µm resin (Dr. Maisch). The analytical column was encased by a column oven (PRSO-V1; Sonation) and attached to a nanospray flex ion source (Thermo). The column oven temperature was adjusted to 45°C during data acquisition. The LC was equipped with two mobile phases: solvent A (0.1% formic acid [FA] in water) and solvent B (0.1% FA in acetonitrile [ACN]). All solvents were of ultra-performance liquid chromatography (UPLC) grade (Sigma-Aldrich). Peptides were directly loaded onto the analytical column with a maximum flow rate that would not exceed the set pressure limit of 980 bar (usually around 0.6–1.0 µL/min). Peptides from IGD were subsequently separated on the analytical column by running a 70-min gradient of solvent A and solvent B (start with 7% B; gradient 7% to 35% B for 60 min; gradient 35% to 80% B for 5 min; and 80% B for 5 min) at a flow rate of 300 nL/min. The mass spectrometer was operated using Xcalibur software (version 2.2 SP1.48). The mass spectrometer was set in the positive ion mode. Precursor ion scanning was performed in the Orbitrap analyzer (Fourier transform mass spectrometry [FTMS]) in the scan range of *m*/*z* 300–1,500 (IGD) or 1,800 (ISD) and at a resolution of 60,000 with the internal lock mass option turned on (lock mass was 445.120025 *m*/*z*, polysiloxane) (38). Product ion spectra were recorded in a data-dependent fashion in the ion trap (ITMS) in a variable scan range and at a rapid scan rate (wideband activation was turned on). The ionization potential (spray voltage) was set to 1.8 kV. Peptides were analyzed using a repeating cycle consisting of a full precursor ion scan (3.0 × 10^6^ ions or 50 ms) followed by 12 product ion scans (1.0 × 10^4^ ions or 80 ms) where peptides are isolated based on their intensity in the full survey scan (threshold of 500 counts) for tandem mass spectrum (MS2) generation that permits peptide sequencing and identification. Collision-induced dissociation (CID) energy was set to 35% for the generation of MS2 spectra. During MS2 data acquisition, dynamic ion exclusion was set to 120 seconds with a maximum list of excluded ions consisting of 500 members and a repeat count of one. Ion injection time prediction, preview mode for the FTMS, monoisotopic precursor selection, and charge state screening were enabled. Only charge states higher than 1 were considered for fragmentation.

### Peptide and protein identification using MaxQuant

For peptide and protein identification, RAW spectra were submitted to an Andromeda (39) search in MaxQuant (1.5.3.30 or) using the default settings (40). Label-free quantification and match-between-runs were activated (41). The Uniprot reference database for *R. leguminosarum* bv. viciae (strain 3841) was used for the search (UP000006575_216596.fasta; 7093 entries; downloaded 06.12.2016). All searches included a contaminants database search (as implemented in MaxQuant, 245 entries). The contaminants database contains known MS contaminants and was included to estimate the level of contamination. Andromeda searches allowed oxidation of methionine residues (16 Da) and acetylation of the protein N-terminus (42 Da) as dynamic modifications and the static modification of cysteine (57 Da, alkylation with iodoacetamide). Enzyme specificity was set to “Trypsin/P” with two missed cleavages allowed. The instrument type in Andromeda searches was set to Orbitrap and the precursor mass tolerance was set to ±20 ppm (first search) and ±4.5 ppm (main search). The MS/MS match tolerance was set to ±0.5 Da. The peptide spectrum matches false discovery rate (FDR) and the protein FDR were set to 0.01 (based on the target-decoy approach). The minimum peptide length was seven amino acids. For protein quantification, unique and razor peptides were allowed. Modified peptides were allowed for quantification. The minimum score for modified peptides was 40. Label-free protein quantification was switched on, and unique and razor peptides were considered for quantification with a minimum ratio count of 2. Retention times were recalibrated based on the built-in nonlinear time-rescaling algorithm. MS/MS identifications were transferred between LC-MS/MS runs with the “match between runs” option in which the maximal match time window was set to 0.7 min and the alignment time window set to 20 min. The quantification is based on the “value at maximum” of the extracted ion current. At least two quantitation events were required for a quantifiable protein. Further analysis and filtering of the results was done in Perseus (42). Comparison of protein group quantities (relative quantification) between different MS runs is based solely on the LFQs as calculated by the MaxQuant MaxLFQ algorithm (41).

### RNASeq

Samples of RNA (in triplicate) were prepared from strains OPS1907, OPS1908, and RU4062 (pK19 mutants in RL3453, RL4145 [*pckR*], and pRL100162 [*nifH*], respectively) (Table S1) grown for 3 days at 28°C on UMS agar slopes. Bacteria were resuspended in 1:1 UMS and RNAlater. RNA extraction was carried out using an RNeasy mini kit (Qiagen), and genomic DNA was depleted using two rounds of removal with the TURBO DNA-free kit (Ambion). Complete removal of DNA was confirmed using a Qubit (BR DNA kit), and RNA quality was assayed using the RNA 6000 Nano kit on a 2100 Bioanalyzer System (Agilent). Sequencing of samples was carried out by Novogene using the Illumina NovoSeq 6000. Analysis was performed as described previously (43) using EDGE-pro v 1.3.1 (44), a pipeline developed for the analysis of prokaryotic RNASeq data. EDGE-pro uses Bowtie2 v 2.3.5 to map reads to the reference genome (*Rhizobium leguminosarum* bv. viciae 3841 reference genome) and calculates the number of reads and TPM values for each feature in the genome. Mapped reads were processed and adjusted *P*-values were calculated using DESeq2. Genes with a greater than or equal to an eightfold change in expression and an adjusted *P*-value < 0.01 were considered significantly differentially expressed. The output of the DESeq2 analysis is provided in Data sheet S3.

### General computing

Geneious R10 was used for primer design and local sequence alignment (45). Global nucleotide and protein sequence alignments were carried out using BLASTn and BLASTp (NCBI) (46). Protein-protein interaction networks were predicted and visualized using STRING (47). Cellular protein localization was predicted using pSORTb v 3.0.2 (48). In bacterial attachment assays, luminescence was evaluated using IndiGO software (Berthold) and subjected to statistical testing using Student’s *t*-test (49) and unpaired *t*-test (50) in GraphPad Prism 8. Data handling was largely in MS Excel and all graphs were generated using GraphPad Prism 8. To discover potential RpoH1- and RpoH2-binding sites in the Rlv3841 genome, sequence searches were carried out using FIMO from the MEME suite of software (51).

## RESULTS

### Lux-based assay for primary attachment of *R. leguminosarum* to pea roots

A Lux-based assay was developed to measure the attachment of bacteria to pea roots after 1 h of incubation. We defined this as primary attachment because we saw no statistically significant difference between the number of bacteria recovered from a pea root (approx. 2 × 10^6^ CFU) by reversible attachment (vortexing roots) and irreversible attachment (vortexing and grinding of roots) (Fig. S2). As we are measuring the attachment of bacteria to roots after 1 h, colony-forming units (CFU) are a good approximation of the number of bacteria.

**Fig 1:**
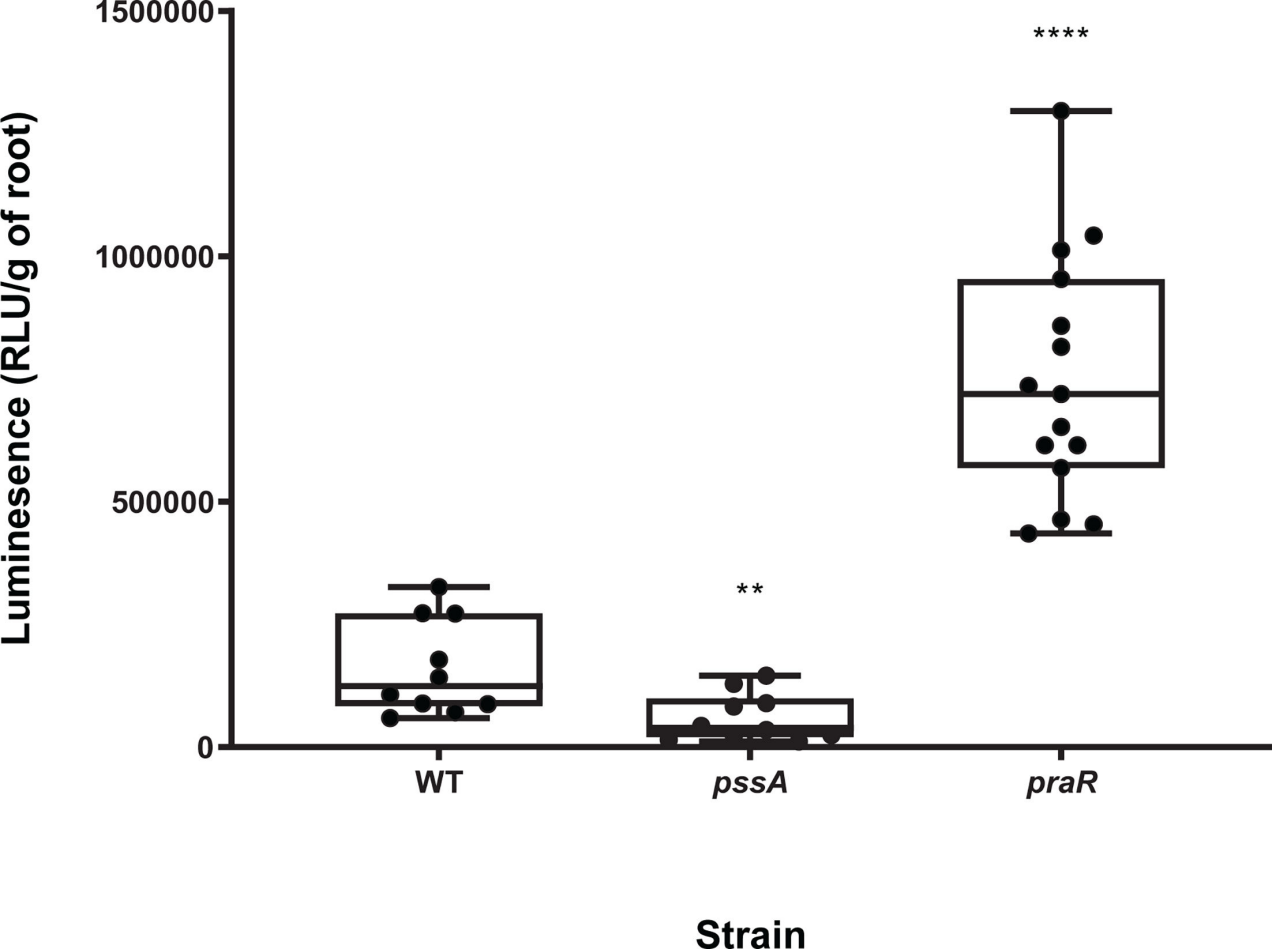
Primary attachment to pea roots of wild-type and mutant strains of *R. leguminosarum* at pH 7.0. Attachment of Rlv3841 (WT) and strains mutated in *pssA* (RL3752) and *praR* (RL0390) was measured using the Lux-based whole root attachment assay where each strain is labeled by the introduction of a plasmid constitutively expressing Lux. Luminescence (RLU/g of root) shows bacterial attachment after 1 h. *n* ≥ 10. All data points are shown with the box indicating the interquartile range and the median shown. Maximum and minimum values are indicted by the whiskers. ****P < 0.0001, **P < 0.01 in comparison to WT using Student’s *t*-test.

Using the Lux-based attachment assay (bacteria constitutively labeled with *luxCDABE*) (13), primary attachment of wild-type (WT) Rlv3841 to pea roots at pH 7.0 was approx. 1.5 × 10^5^ RLU/g of root (Fig. 1). To validate the 1-h attachment assay experimental design, results for strains with mutations in genes previously reported to affect primary attachment were compared with WT (Fig. 1). A *pssA* (RL3752) mutant shows decreased primary attachment (approx. 5 × 10^4^ RLU/g of root, significantly different from WT [*P* < 0.01]) (Fig. 1). The *pssA* gene encodes a glycosyl transferase involved in acidic EPS biosynthesis and a mutant in *pssA* lacks both EPS and capsular polysaccharide (4). A strain mutated in RL0390 (*praR*), a negative transcriptional regulator of several cell surface components, shows increased attachment (approx. 7.5 × 10^5^ RLU/g of root, significantly different from WT [*P* < 0.0001]) (a phenotype initially reported by Frederix et al. [13]). These two mutant strains with altered ability to attach to roots (4, 13) have established that this Lux-based assay works effectively across a range of bacterial primary attachment (both increased and decreased relative to WT).

**Fig 2:**
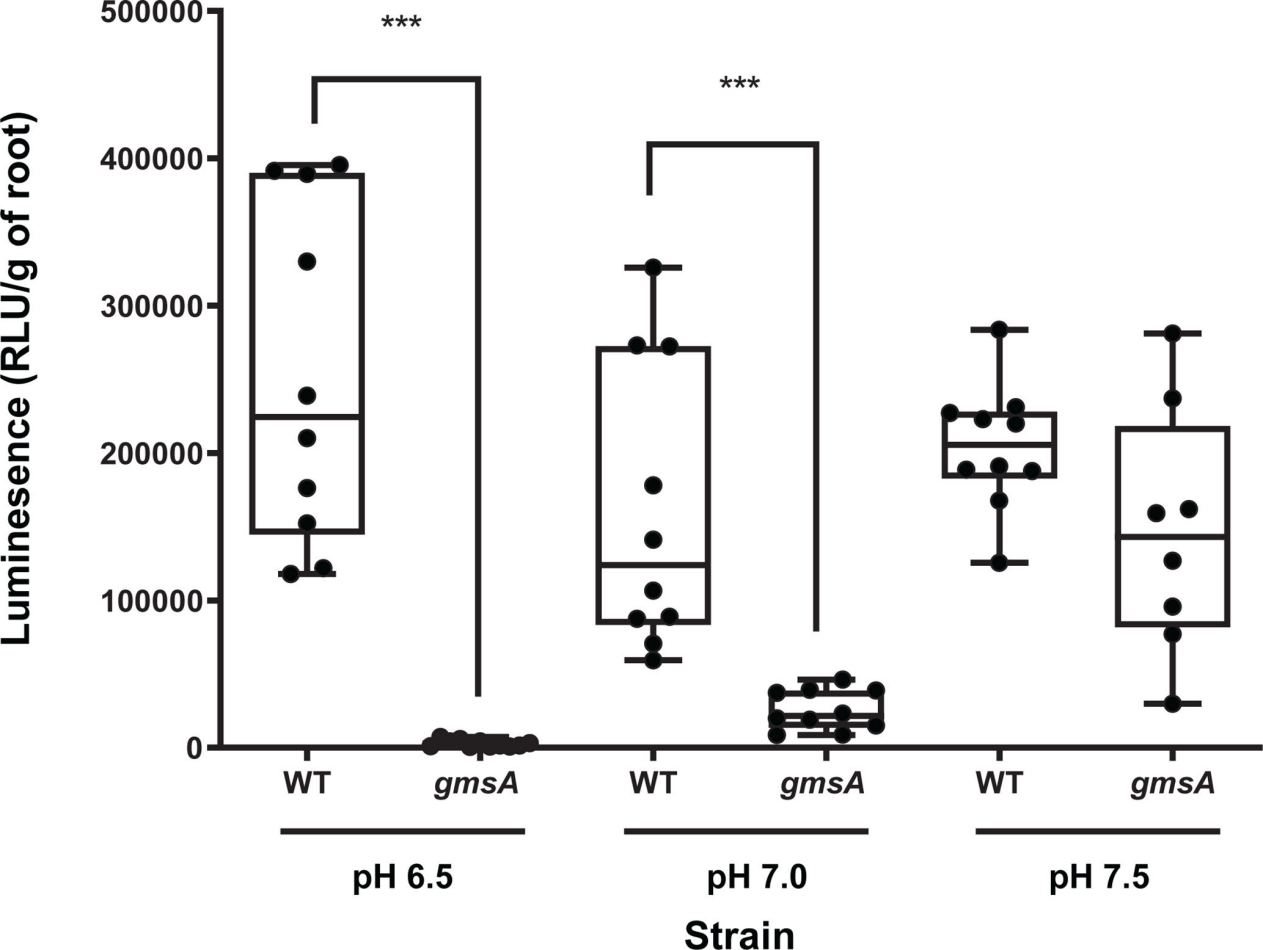
Effect of pH on primary attachment to pea roots. Attachment of Rlv3841 (WT) and a strain mutated in *gmsA* at acidic, neutral, and alkaline pH using the Lux-based whole root attachment assay. Luminescence (RLU/g of root) shows bacterial attachment after 1 h. pH is indicated. *n* ≥ 10. All data points are shown with the box indicating the interquartile range and the median shown. Maximum and minimum values are indicted by the whiskers. ***P < 0.001 using Student’s *t*-test. There is no statistically significant difference between primary attachment to roots of WT at the three different pHs.

While primary attachment of WT to roots was not statistically different at any pH from 6.5 to 7.5 (Fig. 2), a *gmsA* strain, lacking glucomannan (4), had reduced attachment compared to WT (*P* < 0.001) at pH 6.5 and pH 7.0, but not at pH 7.5 (Fig. 2). Therefore, 1-h Lux-based assays for primary attachment (Fig. 1 and 2) agree with previous results for key mutants affected in primary adhesion, including pH profile, and were adopted as the standard conditions to assess primary attachment.

### *mariner* transposon classification of genes affected in primary attachment

Having validated a 1-h attachment assay, INSeq was used to investigate the attachment of Rlv3841 to pea under acid (pH 6.5), neutral (pH 7.0), and alkaline (pH 7.5) conditions. This inoculum density allows for >3,000-fold coverage of the approx. 7,300 Rlv3841 genes (and >165-fold coverage of the 140,056 TA sites). In contrast to INSeq root colonization experiments, involving bacterial growth and survival over a much longer timescale (e.g., 3–7 days post-inoculation [21, 22]) where colonization events are low, there is no such bottleneck in examining primary root attachment. The average input transposon insertion density (% of TA sites carrying one or more insertions) was 82%, while the averages for output densities were 48% (pH 6.5) and 50% (pH 7.0 and pH 7.5). Approximately 97% of the Rlv3841 genome (7,335 genes, encoded on a circular chromosome and six plasmids [present in a single copy] [52]), contain one or more TA sites available for INSeq analysis. Using HMM analysis, considered robust over a range of output densities (32), we classified genes into the following categories: essential (ES) (tolerates no or very few insertions), defective (DE) (insertion impairs fitness), neutral (NE) (insertion has a neutral impact on fitness), or advantaged (AD) (insertion enhances fitness), that is, the same criteria defined for essential (ES), growth defective (GD), neutral (NE), and growth-advantaged (GA), respectively (32), (designations describing growth were considered inappropriate for this study). In the input library, 6,150 genes (87%) were classified as NE. Genes in the input library classified as ES/DE (these two categories together make 11% of genes required in the input library) and AD (3%) (in total 947 genes) are already compromised prior to testing attachment to plant roots and were not considered further in this study. In addition, the following were removed: likely pseudogenes (8) and those non-NE for growth in TY (156) (23), since the bacteria were grown on TY after recovery from the roots. A total of 5,986 genes were assessed for their role in primary attachment (Data sheet S1). Through HMM analysis of primary attachment at each pH (6.5, 7.0, and 7.5), each of the 5,986 genes was assigned an INSeq classification (reproducibility between triplicates 99.0% and 99.4%) (Data sheet S1). While the overall pattern of gene classification is remarkably similar at all pHs: ES 0.1%–0.2%, DE 2.3%–2.5%, NE 96%–97%, and AD <0.01% (with approx. 1% failing to give a clear classification), the genes falling into the categories at each pH are different (Data sheet S1).

**Fig 3:**
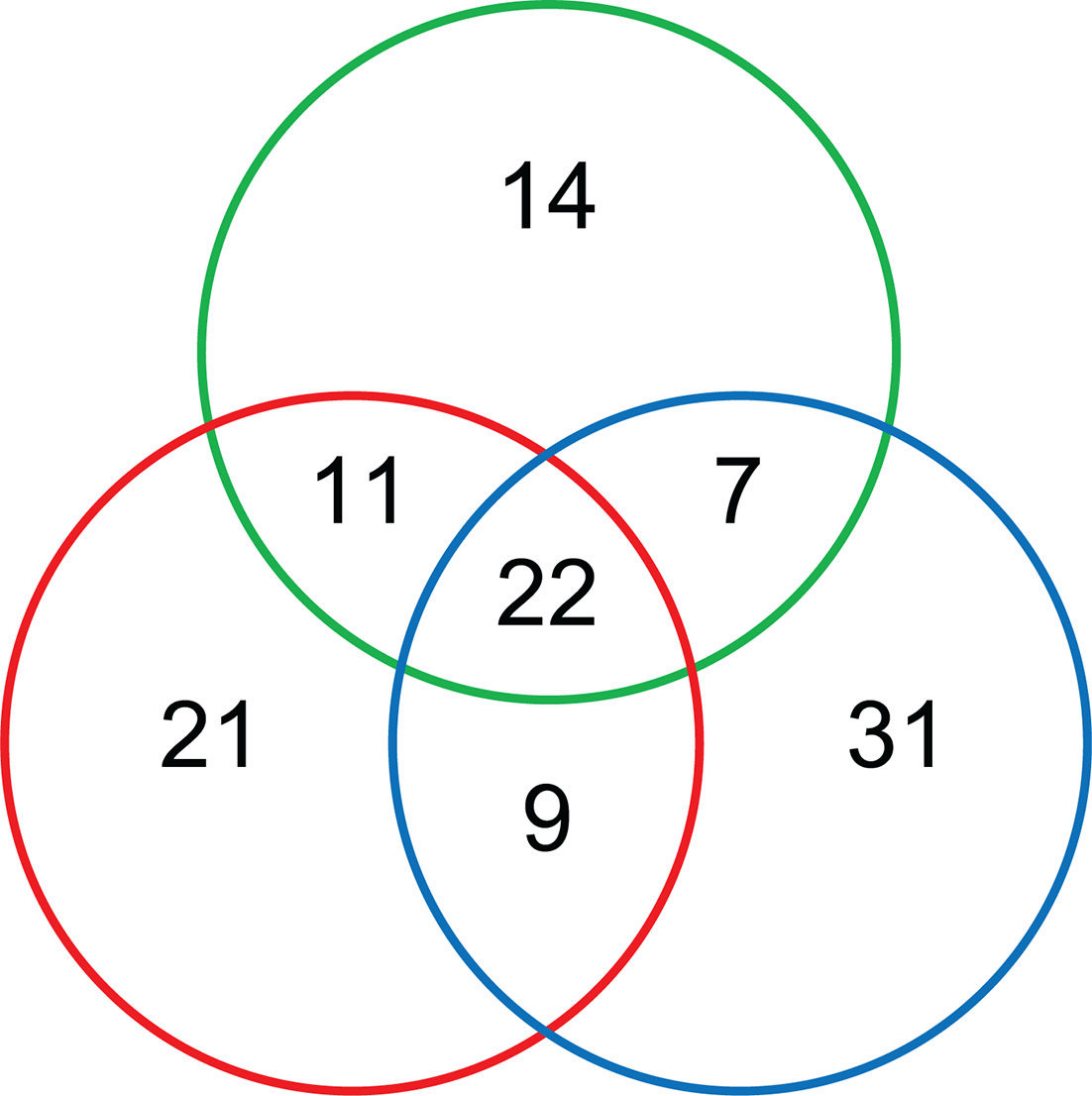
Rlv3841 genes required for primary attachment to pea roots under acidic, neutral, and alkaline pH. A total of 115 genes were classified as required for attachment (ES/DE) in INSeq experiments performed at pH 6.5, pH 7.0, and pH 7.5 (Table S3), with 22 genes being required for attachment at all pHs (Table 1). The color of the circle indicates pH; on the left-hand side (red) = pH 6.5, at the top (green) = pH 7.0, and on the right (blue) = pH 7.5. Genes are listed in Tables S4 to S9.

In total, 280 genes are required for root attachment (grouping those classified ES/DE as “required”) (Data sheet S2). Since we are interested in genes with a specific role in attachment, genes required for growth and survival in previous INSeq analyses (i.e., growth on glucose or succinate at 20% and 3% oxygen [25] and growth in Vincents minimal media containing mannitol [53]) were also removed, leaving 115 genes in the “attachome” (Fig. 3; Table S3). Twenty-two genes are required for attachment at pH 6.5, pH 7.0, and pH 7.5 (Fig. 3; Table 1), with genes required at specific pHs (Fig. 3) listed in Tables S4 to S9.

**TABLE 1:** Core attachome of 22 genes required specifically for primary attachment to pea roots by Rlv3841 at pH 6.5, pH 7.0, and pH 7.5

Gene	Name	Description
pRL100053^*a*^		Putative transmembrane protein with helix-turn-helix 37 domain
pRL100174		Conserved hypothetical protein
RL0551	*hslO*	Hsp33-like chaperonin HslO
RL0876		Conserved hypothetical cytoplasmic protein
RL1381		Uncharacterized protein. Unknown localization
RL1478	*amn*	AMP nucleosidase; catalyzes hydrolysis of AMP to form adenine and ribose 5-phosphate
RL2400^*b*^		Putative MarC family transmembrane protein, unknown function
RL2513	*tpiA*	Putative triosephosphate isomerase TpiA
RL2637	*recA*	DNA repair and SOS response RecA
RL3322	*pfp*	Putative pyrophosphate-fructose 6-phosphate 1-phosphotransferase
RL3752^*a*^	*pssA*	Glycosyl transferase involved in EPS biosynthesis
RL3766	*rpoH1*	RNA polymerase sigma-32-factor, heat shock sigma factor RpoH1
RL3987^*b*^	*vapB*	SpoVT-AbrB domain-containing protein. Antitoxin (VapB) of VapBC TA
RL3988	*vapC*	PIN domain-containing protein. Single-stranded RNA nuclease. Toxin (VapC) of VapBC TA
RL3989	*ruvA*	Holliday junction ATP-dependent DNA helicase RuvA
RL3990	*ruvB*	Holliday junction ATP-dependent DNA helicase RuvB
RL4065^*b*^		Hypothetical cytoplasmic protein with no conserved domains
RL4145^*a*^^,*c*^	*pckR*	LacI family transcriptional regulator
RL4362^*b*^		Putative cobalamin (vitamin B12) synthesis protein, CobW domain
RL4363	*dacC*	Putative penicillin-binding protein, peptidase S11 domain
RL4381		Putative POTRA-domain transporter
RL4382^*a*^		Filamentous hemagglutinin adhesin

^*a*^ Mutant made and strain assessed in Lux-based assay for primary attachment (four genes).

^*b*^ Gene contains fewer than 6 TA sites (four genes).

^*c*^ Gene encodes a regulator of gene transcription. RNASeq was performed on a strain mutated in this gene (one gene).

As the incubation time on roots was 1 h, most of the gene INSeq classifications in this study will not reflect induction by pea roots. For example, even in an optimized temperature-regulated response system in *E. coli*, marked gene induction was not seen in under 2 h (54). Expression of the genes classified as required for attachment is either constitutive or occurs in the bacterial growth conditions prior to the assay (Fig. 3).

### Twenty-two genes required for attachment at all pHs make up the core attachome

**Fig 4:**
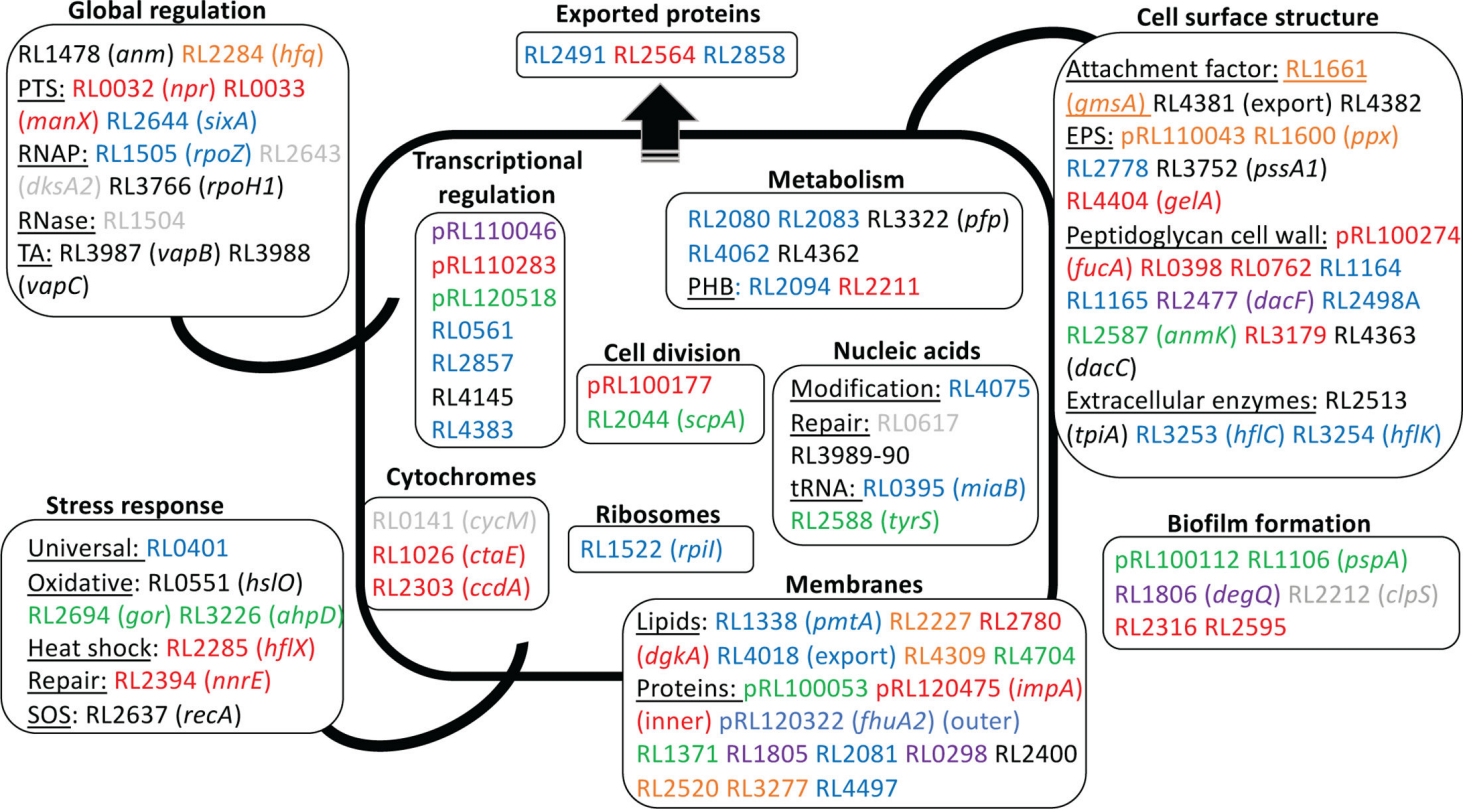
Summary of Rlv3841 genes required for primary attachment to pea roots at acidic, neutral, and alkaline pH grouped by function. From INSeq, the 115 genes required at pH 6.5 to pH 7.5 (Fig. 3; Table S3) are shown grouped by their involvement in different cellular structures or processes, and color-coded. Genes required at all pHs (the core attachome) are shown in black and those required under specific conditions are as follows: pH 6.5 (red), pH 7.0 (green), pH 7.5 (blue), at pH 6.5 and pH 7.0 (orange), pH 7.0 and pH 7.5 (gray), and pH 6.5 and pH 7.5 (purple). In addition, there are 24 genes that have an, as yet, unidentified function (Table S3). Abbreviations: TA, toxin:antitoxin; PTS, phosphotransferase system; RNAP, RNA polymerase; PHB, poly-hydroxy butyrate.

The core “attachome” of 22 genes required at all of the three pH conditions tested (Table 1; Fig. 3 and 4) contains structural components of the cell surface and membranes. These include the cell-surface filamentous hemagglutinin adhesin (RL4382), together with its transporter (RL4381). Also required are enzymes involved in the biosynthesis of surface macromolecules, acidic EPS and capsular polysaccharide (RL3752 [*pssA,* glycosyl transferase]), and peptidoglycan (RL4363 [*dacC*]). Rhizobial mutants in *pssA* are deficient in acidic EPS production, form biofilms slowly compared to WT, and do not attach to root hairs (4). The biofilms formed by *pssA* mutants are flat and unstructured (8).

Other genes in the core attachome (Table 1; Fig. 4) encode proteins involved in adaption to an environment and managing resulting stress, for example, RL0551 (*hslO*), a chaperonin whose function is to protect thermally unfolding and oxidized proteins from aggregation (55) and RL1478 (*amn*), an AMP nucleosidase which catalyzes the hydrolysis of AMP. Changes in AMP levels allow rapid adjustments to changing metabolic conditions (56). Genes involved in DNA repair under changing environments include RL2637 (*recA*) (also responsible for the SOS response), with disruption of *recA* shown to reduce adherence and colonization of host cells by *Vibrio cholerae* (although the mechanism remains unknown [57]) and RL3989-90 (*ruvAB*) encoding Holliday junction ATP-dependent DNA helicases. RuvAB is involved in DNA damage repair and is thought to be involved in the osmotic shock response (58). In addition, DNA-repair systems are involved in dampening the oxidative burst upon recognition of microbial-associated molecular patterns (MAMPs) by the plant immune system receptors. Given the 1-h attachment assay and the time scale of the oxidative burst (within minutes) seen in response to MAMPs (59), this provides a plausible explanation for the requirement of these genes. Located immediately upstream of RL3989 are the overlapping open reading frames RL3987 (encoding a SpoVT-AbrB domain-containing protein of 87 amino acids) and RL3988 (encoding a PIN domain-containing protein of 133 amino acids), which have the characteristics of a toxin-antitoxin (TA) pair of the Virulence-Associated Protein (VapBC) family (60). Often sharing little primary sequence homology other than three specific acidic residues, toxin VapC (RL3988) is an RNase, cleaving single-stranded RNA in a sequence-specific, Mg^2+^- or Mn^2+^-dependent manner (60). Antitoxin VapB (RL3987) renders VapC inactive through protein-protein interactions. A large number of VapBC TA systems are found in many prokaryotes, including *S. meliloti* which has 21 such systems (60). These include NtrPR, which has been shown to regulate a wide range of metabolic transcripts, presumed to mediate symbiosis, and environmental and other stresses (61).

In addition, genes encoding proteins controlling gene transcription, some potentially with a role in adaption and tolerance to hostile/stressful conditions are also required. These include the LacI family transcription factor RL4145 (*pckR*) (further investigated by RNASeq, see below) and the alternative sigma factor (heat shock sigma factor) RL3766 (*rpoH1*) (Table 1). Although the 1-h attachment assay in itself was thought to exclude factors resulting from major changes in gene expression as there simply is not time for their induction, there is evidence for the involvement of *rpoH1*, whose role is to alter transcription under different conditions. Similar to *S. meliloti*, where RpoH1 and RpoH2 share 42% identity (62), Rlv3841 has two RpoHs, encoded by RL3766 (*rpoH1*) and RL4614 (*rpoH2*), with 47% identity (and showing 87% and 75% identity, respectively, with their *S. meliloti* homologs). In *S. meliloti,* it is only RpoH1 that complements mutation of the *E. coli* heat shock sigma factor and only mutation of *rpoH1* leads to a *S. meliloti* Fix- phenotype (62). In Rlv3841, while *rpoH1*, the heat shock sigma factor, is part of the core attachome, *rpoH2* is not required for attachment to roots (Data sheet S1). Sequence motifs identified for genes regulated by RpoH1 and RpoH1/H2 of *S. meliloti* (63) were used to probe the Rlv3841 genome (Tables S10 and S11). Five genes required for root attachment have RpoH1 recognition sequences upstream: pRL100053, *rpoH1* (Tables 1 and 4), RL2520, RL2694 (Table 4), and RL4062 (Tables S3 and S6) (note that RpoH1 regulates its expression). Genes pRL120322 (*fhuA2*) (Tables S3 and S6) and RL4382 (Tables 1 and 4) have the motif for RpoH1/H2 binding. It is highly likely that the reason RpoH1 is required for root attachment is because its mutation disrupts the expression of these genes. It is unknown whether the changes to transcription happened prior to or during the brief 1-h attachment assay. In summary, the core attachome contains genes encoding cell surface and membrane components, together with enzymes responsible for their synthesis and modification. Mechanisms of stress management and dampening the oxidative response to MAMPs, through protein protection, DNA repair, a heat shock sigma factor, and the VapBC TA system, are crucial for primary attachment at all pHs.

### Genes required for root attachment vary with environmental pH

There are substantial differences in the gene requirements for attachment under different pH conditions (Fig. 3). Genes required under specific conditions of acidic and alkali pH, as well as in combination with neutral pH, are listed in Tables S4 to S9. Some of the key genes required and what they reveal overall about bacterial attachment are discussed briefly.

There are 21 genes specifically required for root attachment only at pH 6.5 (Table S4). Among these are RL0032 (*npr*) and RL0033 (*manX*) which encode part of the phosphenolpyruvate phosphotransferase system (PTS) (64). Mutants in PTS genes have a “dry” morphology due to reduced secretion of EPS (65). Perhaps, crucially at low pH, reduction in surface EPS has a greater effect on the bacterial surface, leading to a drop in the ability to attach to roots. Likewise, there is a requirement for pRL100274 (*fucA*), which encodes an α-L-fucosidase that cleaves fucosidic bonds in glycans (particularly in peptidoglycan structures) (66) and is likely involved in remodeling of the cell surface. Also required are RL0398, encoding a putative N-acetyltransferase, which may be involved in peptidoglycan modification, RL0726 (putative transglycosylase) able to degrade peptidoglycan via β 1–4 glycosidic bond cleavage (67), RL3179, encoding a putative cobalamin (vitamin B12) synthesis protein (which may be involved in peptidoglycan amidation), and RL4404 (*gelA*) encoding gel-forming EPS production protein (4). A requirement for these genes, as well as those for membrane proteins, suggests the importance at pH 6.5 for both the presence and the structure of peptidoglycan, found on the cell surface, making up the bacterial cell wall. At acidic pH, plant lectins are present on roots, while at alkali pH they dissociate (6).

Fourteen genes are required specifically only at pH 7.0, including membrane protein RL1371 (Table S5). Thirty-one genes are needed for root attachment specifically at pH 7.5 and include ω subunit of RNA polymerase RpoZ (RL1505) with a potential role in biofilm formation (68, 69) (Table S6). As with genes required for attachment at pH 6.5, the importance of the bacterial cell surface is apparent, as again genes encoding proteins involved in peptidoglycan metabolism are required (these include RL2489A, encoding a transglycosylase-associated protein, and RL2778, encoding an exopolysaccharide biosynthesis protein). Also required is RL2644 (*sixA,* the only known bacterial phosphohistidine dephosphorylase), part of the Npr system that acts to dephosphorylate Npr in *E. coli* (70) and is implicated in biofilm formation (71). Interestingly, Npr itself is required for attachment at pH 6.5 (Table S4), while a lack of SixA, which will leave Npr phosphorylated, is detrimental to bacterial attachment at pH 7.5. One possibility is that phosphorylated Npr inhibits attachment at pH 7.5 but promotes it at pH 6.5. At pH 7.5, we again observe that the composition of membranes is important; RL1338 (*pmtA*), a phosphatidylethanolamine N-methyltransferase involved in membrane lipid phosphatidylcholine synthesis is required.

Genes required at pH 6.5 and pH 7.0 include RL1661 (*gmsA*), RL1600 (*ppx*) an exopolyphosphatase, putative transport proteins pRL110043 and RL2520, and membrane proteins RL3277 and RL4309 (Table S7). Glucomannan, whose synthesis requires *gmsA* (encoding glucomannan synthetase), is well documented to be required for attachment only under acid and neutral conditions and not in an alkaline environment (4). Our INSeq results for *gmsA* of DE at pH 6.5 and pH 7.0, and NE at pH 7.5 (Table 2) are in complete agreement with the reported phenotype (4). In addition, we found the mutation of *gmsA* prevents bacteria from attaching to pea roots at pH 6.5 and pH 7.0 (*P* < 0.0005), but the attachment is not significantly affected at pH 7.5 (Fig. 2; Table 2). Genes required at pH 7.0 & pH 7.5 or pH 6.5 & pH 7.5 are listed in Tables S8 and S9, respectively. The clear conclusion from examining the requirements at different conditions is that the cell surface (composition and structure of both peptidoglycan layer and membranes) is crucially important for effective bacterial attachment to plant roots, although specific requirements depend on the environment.

**TABLE 2:** Comparison of INSeq classification of gene and primary attachment to roots of a strain with the gene mutated^*g*^

Gene	pH 6.5		pH 7.0		pH 7.5		Agreement from the two experimental approaches
	INSeq classification	Primary root attachment (% of WT^*a*^) of strain with gene mutated	INSeq classification	Primary root attachment (% of WT^*a*^) of strain with gene mutated	INSeq classification	Primary root attachment (% of WT^*a*^) of strain with gene mutated	
pRL100053^*b*^^,*c*^	DE	4%***	DE	7%***	DE	19%***	3/3
pRL100162 (*nifH*)	NE	102%	NE	107%	NE	99%	3/3
pRL110071^*d*^	DE	28%***	DE	33%**	DE	20%***	3/3
pRL110543	NE	105%	NE	148%	NE	114%	3/3
RL0109^*d*^	DE	8%***	ES	23%***	ES	18%***	3/3
RL0390 (*praR*)	NE	61%*	NE	467%***	NE	67%**	0/3
RL1661 (*gmsA*)^*c*^	DE	1%***	DE	16%***	NE	71%	3/3
RL2969	NE	8%***	NE	16%***	NE	67%**	0/3 (3/3)^*f*^
RL3273	NE	138%	NE	45%*	NE	42%***	1/3
RL3453^*d*^^,*e*^	DE	30%***	DE	38%**	DE	23%***	3/3
RL3752 (*pssA*)^*b*^^,*c*^	DE	21%***	DE	37%**	DE	38%***	3/3
RL4145 (*pckR*)^*b*^^,*c*,*e*^	DE	0%***	DE	2%***	DE	2%***	3/3
RL4382^*b*^^,*c*^	DE	3%***	DE	20%***	DE	8%***	3/3
						**Total:**	31/39 (34/39)^*f*^

^*a*^ WT is Rlv3841. Results expressed as mean root attachment of test strain in percent indexed to 100% attachment for WT under each condition.

^*b*^ Gene listed in Table 1, which shows the 22 genes required (classified ES/DE) specifically for attachment at pH 6.5, pH 7.0, and pH 7.5.

^*c*^ Gene listed in Table 4, which shows the 54 genes required for attachment at pH 7.0.

^*d*^ Gene does not appear in Table 1 since not specific for attachment. pRL110071, RL0109, and RL3453 were classified ES or GD in INSeq performed under one or more growth conditions (23, 25, 53).

^*e*^ Gene encodes a regulator of gene expression. RNASeq was performed on a strain mutated in this gene.

^*f*^ Mutant able to attach to pea roots at a level not significantly different from that of WT, when inoculated at a ratio of mutant:WT of 1:100 (Fig. 6).

^*g*^ INSeq classification abbreviations: ES essential, DE defective, NE neutral. Asterisk signifies a statistically significant difference from WT using an unpaired *t*-test. *P < 0.05, **P < 0.01, ***P < 0.0005.

### Importance of genes required for attachment in other steps of symbiosis

**Fig 5:**
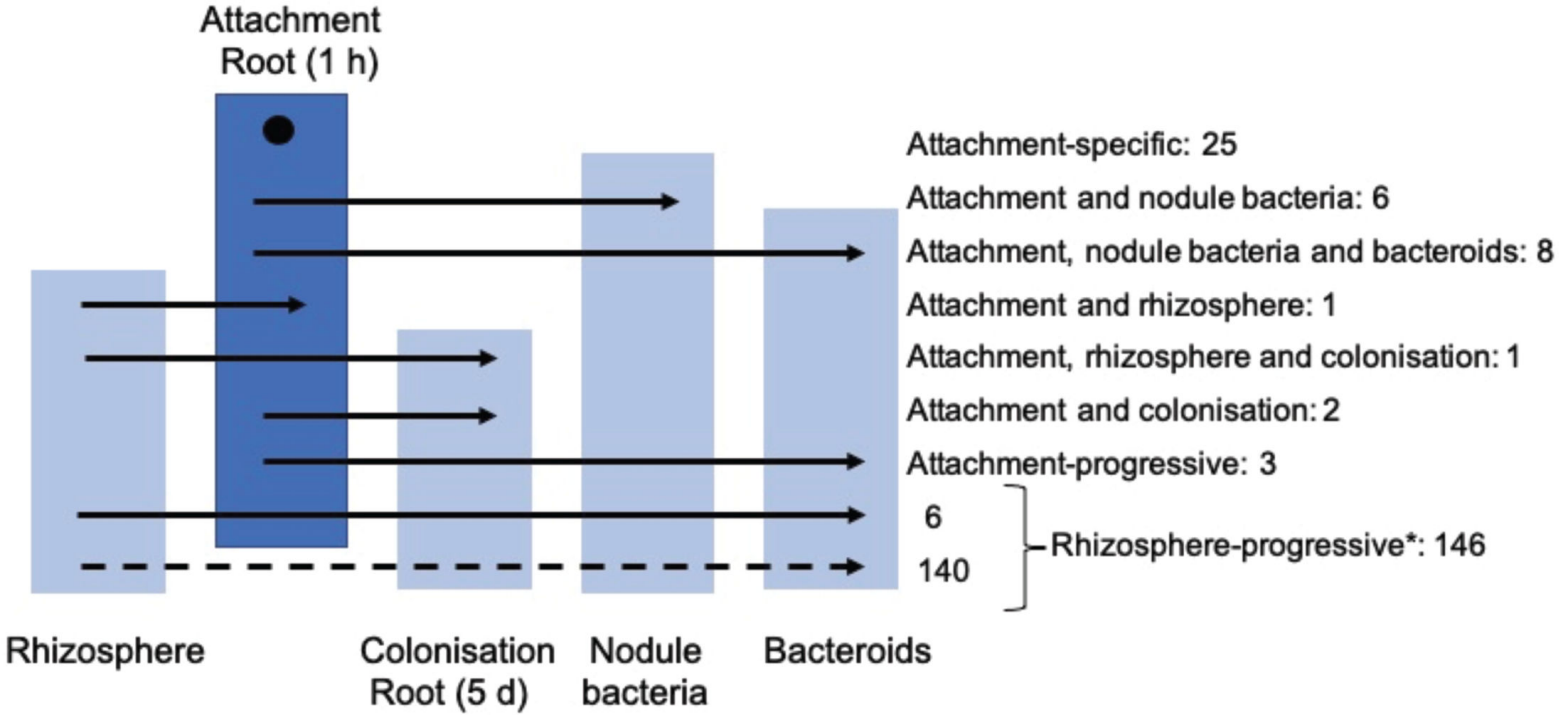
Involvement of genes required for root attachment at pH 7.0 at other stages of nodule development (in the rhizosphere, for root colonization, in nodule bacteria and bacteroids). Fifty-four genes are required for primary root attachment (Fig. 3) and their involvement at other stages of infection and symbiosis was determined from the INSeq study of nodule development (26). Twenty-five genes are root-attachment specific, with the remainder required at one or more stages of symbiosis (attachment to roots (1 h) is represented by a dark blue box, while those of other developmental stages are pale blue). Two genes (RL2400 and RL2642) are not represented; they are INSeq-classified as AD in nodule bacteria (26) (Table 3). *Rhizosphere-progressive genes, in total 146 (26), with six of them also required for primary attachment.

Comparison of all 54 genes required for attachment at pH 7.0 with the data of Wheatley et al. (26) shows approximately half are crucial at other stages of rhizobial symbiosis (Table 3; Fig. 5). As the series of INSeq experiments investigating genes required from rhizosphere to symbiosis were performed under neutral conditions (26), only attachment at pH 7.0 was considered. Approximately half of the 54 genes are specific for primary attachment and have no further effect on the development of nitrogen-fixing bacteroids (Table 3; Fig. 5). These include RL1661 (*gmsA*), which is classified as NE throughout nodule development (26) (Table 3), and illustrates that while *gmsA* plays a role in primary attachment at pH 7.0, it does not alter nodulation competitiveness which proceeds via root hair attachment.

**TABLE 3:** Genes required for root attachment at pH 7.0 grouped by their requirement at different stages (rhizosphere, root colonization, nodule bacteria, and bacteroids) of nodule development

Gene	Name	Description	pH at which gene is required for attachment
Attachment specific (not required at any other stage)			
pRL100112		Putative dehalogenase-hydrolase (HAD)	pH 7.0
pRL100174		Conserved hypothetical protein	pH 6.5, 7.0, and 7.5
pRL110043		Putative transmembrane arabinose efflux permease transporter protein	pH 6.5 and 7.0
RL0551	*hslO*	Hsp33-like chaperonin HslO	pH 6.5, 7.0, and 7.5
RL0617		Putative dTTP/UTP pyrophosphatase	pH 7.0 and 7.5
RL0876		Conserved hypothetical cytoplasmic protein	pH 6.5, 7.0, and 7.5
RL1052		Uncharacterized protein. Unknown localization	pH 7.0
RL1106^*a*^	*pspA*	Putative PspA (phage shock protein A) family regulator	pH 7.0
RL1381		Uncharacterized protein. Unknown localization	pH 6.5, 7.0, and 7.5
RL1504		Uncharacterized cytoplasmic protein, NYN domain. Possibly novel RNAse with regulatory role	pH 7.0 and 7.5
RL1661^*b*^	*gmsA*	Glucomannan biosynthesis protein GmsA	pH 6.5 and 7.0
RL2044	*scpA*	Segregation and condensation protein A participates in chromosomal division	pH 7.0
RL2513	*tpiA*	Putative triosephosphate isomerase TpiA	pH 6.5, 7.0, and 7.5
RL2520		Permease, transmembrane protein	pH 6.5 and 7.0
RL2587	*anmK*	Anhydro-N-acetylmuramic acid kinase AnmK. Required for cell wall recycling	pH 7.0
RL2695^*a*^		Hypothetical protein (contiguous with RL2694 (*gor*) which is rhizosphere-progressive)	pH 7.0
RL3226	*ahpD*	Alkyl hydroperoxide reductase AhpD	pH 7.0
RL3277		Putative transmembrane DedA family protein	pH 6.5 and 7.0
RL3752^*b*^	*pssA*	Glycosyl transferase involved in EPS biosynthesis	pH 6.5, 7.0, and 7.5
RL4083		Uncharacterized protein, SGHN family esterase domain. Unknown localization	pH 7.0 and 7.5
RL4145^*b*^^,*c*^	*pckR*	LacI family transcriptional regulator. Cytoplasmic protein.	pH 6.5, 7.0, and 7.5
RL4335		Uncharacterized protein	pH 6.5 and 7.0
RL4381		Putative POTRA-domain transporter	pH 6.5, 7.0, and 7.5
RL4382^*b*^		Filamentous hemagglutinin adhesin	pH 6.5, 7.0, and 7.5
RL4704		Putative glyoxylase family protein, member of the VOC superfamily	pH 7.0
Required in attachment and rhizosphere only			
RL2284	*hfq*	RNA-binding protein Hfq. Global post-transcriptional regulator	pH 6.5 & 7.0
RL4065^*a*^^,*d*^		Hypothetical cytoplasmic protein with no conserved domains	pH 6.5, 7.0, and 7.5
Required in attachment and root colonization only			
pRL100242		Uncharacterized cytoplasmic protein	pH 6.5 and 7.0
RL1371^*a*^		Putative transmembrane protein	pH 7.0
Attachment-progressive			
pRL100053^*b*^^,*e*^		Putative transmembrane protein with helix-turn-helix 37 domain	pH 6.5, 7.0, and 7.5
RL1478	*amn*	AMP nucleosidase; catalyzes hydrolysis of AMP to form adenine and ribose 5-phosphate	pH 6.5, 7.0, and 7.5
RL3987^*a*^	*vapB*	SpoVT-AbrB domain-containing protein. Antitoxin (VapB) of VapBC TA	pH 6.5, 7.0, and 7.5
Rhizosphere-progressive (required at every stage)			
RL0052		Uncharacterized protein. Unknown localization	pH 7.0
RL1600	*ppx*	Putative exopolyphosphatase Ppx. Hydrolysis of EPS	pH 6.5 and 7.0
RL2694	*gor*	Glutathione reductase Gor (contiguous with RL2695 which is attachment-specific)	pH 7.0
RL3988	*vapC*	PIN domain-containing protein. Single-stranded RNA nuclease. Toxin (VapC) of VapBC TA	pH 6.5, 7.0, and 7.5
RL3989	*ruvA*	Holliday junction ATP-dependent DNA helicase RuvA	pH 6.5, 7.0, and 7.5
RL3990	*ruvB*	Holliday junction ATP-dependent DNA helicase RuvB	pH 6.5, 7.0, and 7.5
Required in attachment, nodule bacteria, and bacteroids			
pRL120518^*c*^		Putative TetR family transcriptional regulator	pH 7.0
RL0141	*cycM*	Membrane-bound cytochrome c CycM	pH 7.0 and 7.5
RL2212	*clpS*	ATP-dependent Clp protease adaptor protein ClpS	pH 7.0 and 7.5
RL2588	*tyrS*	Tyrosine-tRNA ligase TyrS. Catalyzes attachment of tyrosine to tRNA	pH 7.0
RL2637	*recA*	DNA repair and SOS response RecA	pH 6.5, 7.0, and 7.5
RL4309		Putative transmembrane DedA family protein	pH 6.5 and 7.0
RL4362^*a*^		Putative cobalamin (vitamin B12) synthesis protein, CobW domain	pH 6.5, 7.0, and 7.5
RL4363	*dacC*	Putative penicillin-binding protein, peptidase S11 domain	pH 6.5, 7.0, and 7.5
Required in attachment and nodule bacteria only			
pRL100220		Uncharacterized protein	pH 6.5 and 7.0
RL1013		Uncharacterized protein, 17 kDa Anti 2 motif. Unknown localization	pH 7.0
RL2227		Zinc metalloprotease. Potential role in membrane lipid processing	pH 6.5 and 7.0
RL2643	*dksA2*	Putative DnaK suppressor protein DksA2. Modulates RNA polymerase activity. Cytoplasmic	pH 7.0 and 7.5
RL3322	*pfp*	Putative pyrophosphate-fructose 6-phosphate 1-phosphotransferase	pH 6.5, 7.0, and 7.5
RL3766^*a*^	*rpoH1*	RNA polymerase sigma-32-factor, heat shock sigma factor RpoH1	pH 6.5, 7.0, and 7.5
Required in attachment and advantaged when mutated in nodule bacteria			
RL2400^*a*^^,*f*^		Putative MarC family transmembrane protein, unknown function	pH 6.5, 7.0, and 7.5
RL2642^*a*^		Uncharacterized protein	pH 7.0 and 7.5

^*a*^ Gene contains fewer than 6 TA sites (eight genes).

^*b*^ Mutant made and assessed in Lux-based assay (five genes).

^*c*^ Gene encodes a regulator of gene expression (two genes).

^*d*^ Gene is also required in root colonization (one gene).

^*e*^ Mutant shown to have reduced root colonization (5 dpi) (26) (one gene)

^*f*^ Gene is also required in bacteroids (one gene).

Some attachment genes are also required in the rhizosphere and/or colonization of pea roots (at 5 dpi). The global post-transcriptional RNA-binding protein Hfq (RL2284) is required for bacterial survival in the rhizosphere as well for attachment, but at no other stages of nodule formation, including root colonization (Table 3). In *S. meliloti*, deletion of *hfq* delays nodulation and reduces competitiveness for attachment to alfalfa roots (72).

Employing terminology used by Wheatley et al. (26), there are groups of genes that are “progressive” (needed at this and all subsequent stages) in the process of nodule development. We can consider that primary attachment forms a stage between the rhizosphere and 5-day root colonization (Fig. 5). Gene pRL100053 (encoding a putative transmembrane protein) was classified as colonization-progressive and a mutant was unable to colonize roots (26). Here we show it is, in fact, affected earlier in the developmental process and should be considered attachment-progressive (Table 3), as shown by both INSeq designation and the Lux attachment assay in which a pRL100053 mutant shows only 4%–19% root attachment of WT bacteria (Table 2).

Six bacterial genes required for attachment were shown to be required for nodulation (Table 3; Fig. 5), falling into the group described as rhizosphere-progressive (26). They include the three gene clusters RL3988, *ruvAB* which encodes proteins concerned with DNA repair associated with bacterial stress, and RL0052 (*ppx*) which encodes an exopolyphosphatase likely to be involved in the hydrolysis of EPS. RL1600 (*gor*) encodes a glutathione reductase, which plays a role in the cellular control of reactive oxygen species (Table 3).

Two further groups of genes required for attachment at pH 7.0 are also needed at other stages of the nodulation process, either in both bacteroids and nodule bacteria (eight genes) or only in nodule bacteria (six genes) (Table 3). While it is clear that primary root attachment is not the same as attachment to root hairs (a critical step in the nodulation pathway leading to root curling and engulfment of the bacteria), primary root attachment may be important for bacterial competition and long-term bacterial survival in soil. In summary, approximately half of the genes required for primary attachment at pH 7.0 are specific for primary attachment, while the other half are required in one or more other steps in nodule development leading to successful nitrogen fixation in symbiosis.

### Assessment of INSeq classification of genes using the Lux-based attachment assay

For a selection of genes, the comparison was made between their INSeq classification and the effect of their mutation on bacterial root attachment. Strains made by mutating individual INSeq-identified ES/DE genes pRL100053, pRL110071, pRL110543, RL0109, RL2969, RL3273, RL3453, *pckR,* and RL4382 were assessed for root attachment (Table 2). Results are shown together with those for the control strains mutated in; pRL100162 (*nifH*) (28) (negative control, unaffected in attachment), *praR*, and *pssA* (positive controls with increased and decreased attachment, respectively [8, 13]). Candidate genes for mutation were selected based on their function (e.g., the hemagglutinin encoded by RL4382 and likely to be an attachment factor, transcriptional regulators RL3453 and *pckR*, etc.) and partly at random. While it would have been ideal to have a range of different attachment phenotypes across the pH range, for some genes selected for mutation, it proved difficult and/or impossible to make the mutant (often for trivial reasons, which include one or more of the following technical issues: failure to get a single PCR product of the expected size, failure to obtain a correct clone in *E. coli* [size of cloned product, expected antibiotic resistance etc.], inability to transfer the plasmid from *E. coli* into *R. leguminosarum*, and failure to convincingly map the insertion with PCR mapping primers). In the interest of time, those mutants which had been successfully constructed were analyzed in the Lux whole root assay. The results for each pH are shown together and for each gene, the degree of agreement between the two experimental approaches over all three pHs is included. The negative control strain mutated in *nifH* has WT attachment levels (Table 2), showing that a mutation in a gene not related to primary attachment has no effect. The *praR* mutant shows increased attachment (> 4-fold WT) specifically at pH 7.0 (Table 2), in agreement with the conclusion from other work (13)) and the *pssA* mutant shows 21%–38% of WT attachment (*P* < 0.01) at pH 6.5 and pH 7.5 (Table 2). These levels of observed attachment are in complete agreement with the INSeq classification of NE for attachment of *nifH* and DE for attachment of *pssA*. Results for *praR* are discussed in more detail below.

Under each of the three conditions tested (pH 6.5, pH 7.0, and pH 7.5), there is a total agreement between INSeq-classification of the gene and root attachment by six mutant strains (mutations in pRL100153, pRL110071, RL0109, RL3453, *pckR,* and RL4382) (Table 2). Furthermore, there is an agreement in the INSeq classification of *gmsA* (DE at pH 6.5 and pH 7.0, NE at pH 7.5) and root attachment data (Table 2), which is important, as, unlike the other experimental examples, this is different depending on the environmental pH.

While *praR* is INSeq-classified as NE, its mutation affects primary attachment (Table 2). A mutation in *praR* was already documented as increasing attachment at pH 7.0 (13) and is confirmed by results in this work. We should therefore regard the NE INSeq classification as a false-negative result; INSeq classifies the gene as NE (unaffected for attachment) and yet we show the mutant is affected in attachment in the Lux assay. The suggested minimum number of TA sites for confident assignment in HMM analysis is six (73, 74) and *praR* contains four TA sites, already indicating that predictions made from the INSeq results may not be fully reliable for this gene. While we excluded genes that lack TA sites completely, we did not exclude those with less than six TA sites, but, as shown by this example of *praR*, a degree of skepticism should be used when interpreting results for genes with a low number of TA sites (TA sites within each gene is shown in Data sheets S1 and S2).

**Fig 6:**
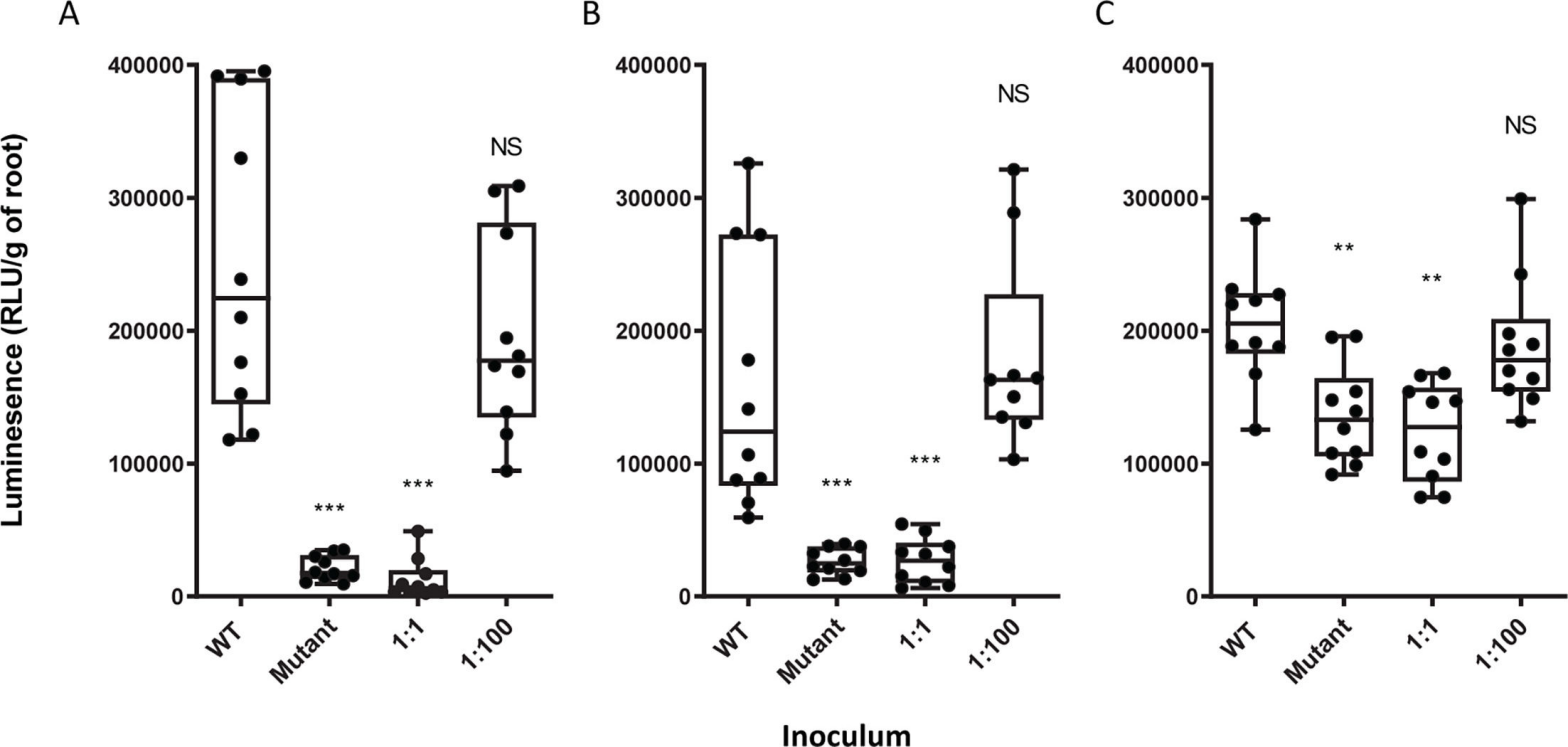
Effect of co-inoculation with wild-type bacteria on primary attachment to pea roots of a mutant at different pHs. Comparison of Rlv3841 (WT) and a strain mutated in RL2969, as single inoculum (Mutant), or in 1:1 or 1:100 co-inoculation with unlabeled Rlv3841 (Mutant:WT) using the Lux-based whole root attachment assay at (**A**) pH 6.5, (**B**) pH 7.0, and (**C**) pH 7.5. Luminescence (RLU/g of root) shows bacterial attachment after 1 h. *n* ≥ 9. All data points are shown with the box indicating the interquartile range and the median shown. Maximum and minimum values are indicated by the whiskers. ***P < 0.001, **P < 0.005 using Student’s *t*-test.

To examine further why there is not complete agreement between the two experimental techniques, we investigated further RL2969 and RL3273 (Table 2). The genes have 23 and 21 TA sites, respectively (Data sheet S1), and are INSeq-classified as NE, but a mutant strain shows reduced attachment. It is pertinent to examine closely and compare what each experiment is assessing; in the Lux-based root assays, attachment of a single mutant strain, alone, is quantified and compared to that of WT, while the INSeq library has approximately 115,000 (82% insertion density of 140,845 genomic TA sites) mutant strains present at the same time, competing to attach to roots. Some mutants will be complemented *in trans* by diffusible factors, or aided in biofilm formation (e.g., by surface polysaccharide). To test if differences in experimental setup have given an apparent discrepancy in results, a Lux-labeled mutant (RL2969 or RL3273 [Table 2]) was assessed for its ability to attach to roots in the presence of unlabeled WT bacteria (at ratios of WT:mutant of 1:1 and 100:1). The strain mutated in RL2969 regained the ability to attach to roots in the presence of a 100-fold excess of WT (Fig. 6) at all pHs. As RL2969 encodes a putative transmembrane protein which is unlikely to be released from the surface of WT cells, it might act to aggregate WT and mutant bacteria on the root surface, thereby complementing the mutant’s attachment-deficient phenotype.

RL3273 encodes a von Willebrand factor type A (VWA) domain-containing protein, implicated in cell adhesion and intracellular enzyme activity in eukaryotes, and widely conserved (75). While the RL3273 mutant’s attachment was not rescued by even a 100-fold excess of WT bacteria at pH 7.0 and 7.5 (Fig. S4) (at pH 6.5, the NE classification agrees with the attachment phenotype, Table 2), this is still a much lower ratio than the 115,000-fold excess present in INSeq experiments. It may be that a far higher ratio of WT cells is required to restore attachment in the RL3273 mutant at neutral and acidic pH. In summary, the results of the two experimental techniques to assess primary attachment are in very good agreement.

### Exploring the role of regulators required for attachment using RNASeq

RNASeq was performed to find the target genes of the only two regulators of expression required at all pHs for primary root attachment: LacI-family transcriptional regulator *pckR* (Table 1) and RL3453 (encoding the histidine kinase of a two-component sensor/regulator) (required for primary attachment but also required under other growth conditions [23, 25, 53]) (Table 2). INSeq-classified as “required” for primary attachment, the role of RL3453 and *pckR* was confirmed by the whole root Lux assay (Table 2). RNASeq was performed on strains OPS1907 and OPS1908 (mutations in RL3453 and *pckR*, respectively), together with strain RU4062 (*nifH* mutant, unaffected for attachment) (Table 2). Many genes are differentially regulated (adjusted *P*-value < 0.01, fold change > 8) compared to the control, especially in OPS1908 (33 up- and 331 genes downregulated), while OPS1907 has no genes upregulated and 148 genes downregulated (Data sheet S3).

In OPS1908 (*pckR* mutant), the most highly upregulated genes are enzymes (*zwf1-pgl-edd* [RL0753-51]) of the Entner-Doudoroff pathway used in *Rhizobiaceae* for the synthesis of glucose. PckR shows 89% identity to that of *S. meliloti*, where it is a key regulator of central carbon metabolism (76). In *S. meliloti,* PckR acts as a repressor of the Entner-Doudoroff pathway (*zwf-pgl-edd*) and an inducer of *pckA, fbaB,* and *mgsA,* genes involved in gluconeogenesis (76). In addition to differential regulation of *zwf1-pgl-edd*, the *R. leguminosarum pckR* mutant shows slight upregulation of *eda2* (RL4162), with concomitant downregulation of *pckA* (RL0037) and *fbaB* (RL4012), which mirrors *S. meliloti* (76). Expression of *mgsA* (RL0183, approx. 2.8-fold up) contrasts with *S. meliloti* where PckR acts as a positive regulator (76). Many genes differentially regulated in this *R. leguminosarum pckR* mutant encode integral membrane proteins, exported proteins, transport system components (both uptake and export), and enzymes (e.g., exopolysaccharide metabolism). It seems unregulated carbon metabolism resulting from mutation of the key regulator PckR leads to widespread changes likely to affect both the cell surface and compounds transported across it. We found far more genes differentially regulated in the *R. leguminosarum pckR* mutant than was observed for *S. meliloti* (76). This may be due to the carbon source used to grow the bacteria (pyruvate in the case of *R. leguminosarum*, compared to glucose or succinate in *S. meliloti* [76]).

In strain OPS1907 (RL3453 mutant), as in OPS1908, the gene encoding transmembrane protein RL0933 is the most highly downregulated. With over 90% of the downregulated genes in OPS1907 (138 of 148), also downregulated in OPS1908 (Data sheet S3), the genes affected in these two mutants show a huge overlap. We conclude that rather than controlling the expression of a specific attachment factor, mutation of *pckR* or RL3453 causes widespread disruption and affects the whole bacterial surface, leading to the observed reduction in root attachment (Table 2).

### Investigation of the gene encoding rhicadhesin

**Fig 7:**
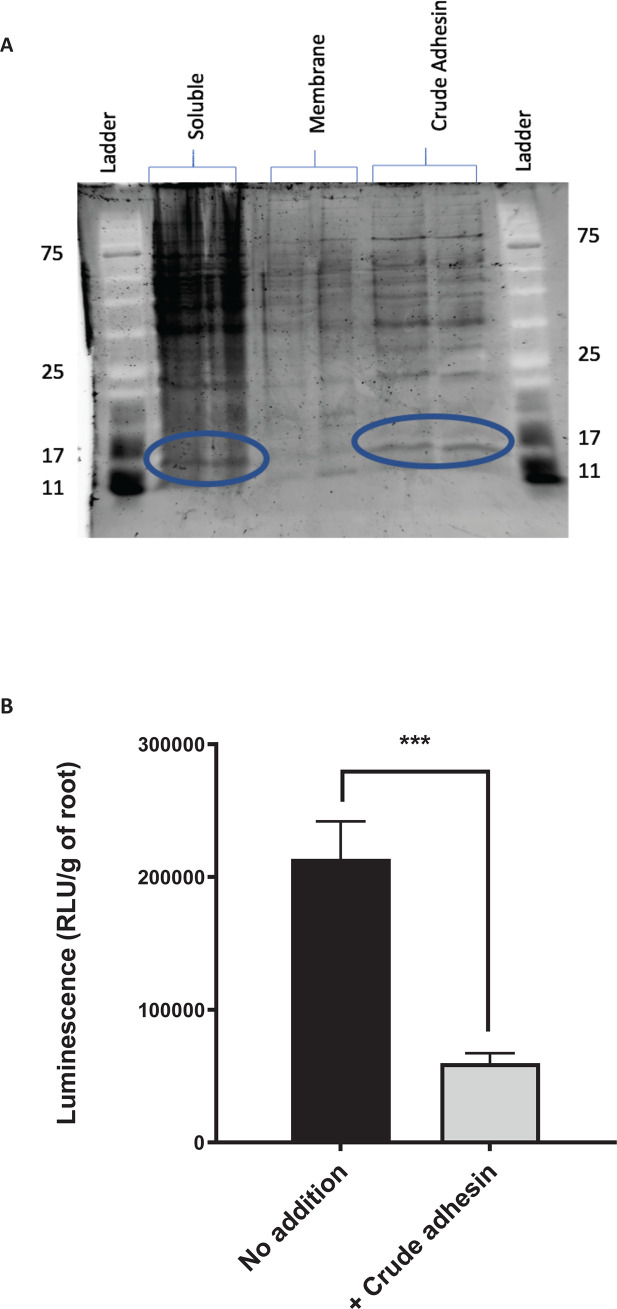
Purification of a crude adhesin protein fraction and its effect on primary attachment of Rlv3841 (WT) to pea roots. (**A**) SDS-gel of Rlv3841 cell fractions (soluble, membrane, and crude adhesin) stained with SYPRO Ruby. The putative rhicadhesin fraction, a protein band at approx. 14 kDa (circled in blue), is visible in both soluble and crude adhesin fractions, the latter of which was excised from the gel and used for proteomic analysis and pre-incubation of roots. Ladder protein sizes (kDa) are indicated. (**B**) Root attachment of Rlv3841 (WT) using the Lux-based whole root attachment assay at pH 7.5 either with no addition (black) or pre-incubation of roots with crude adhesin for 1 h (+ Crude adhesin, gray). Luminescence (RLU/g of root) shows bacterial attachment after 1 h. Data are displayed as mean ± SEM, *n* = 5. ***P < 0.001 using Student’s *t*-test.

In an attempt to identify the gene encoding rhicadhesin (33), we performed cell fractionation and isolation of protein fractions as previously described in the original work (33) (Fig. 7A). The crude adhesin fraction was isolated from the protein gel by excising the band running at approx. 14 kDa (Fig. 7A). Following pre-incubation of pea root sections with the crude adhesin protein fraction, attachment of WT Rlv3841 was significantly reduced (Fig. 7B).

Analysis by LC-MS of the crude adhesin fraction isolated contained 15 proteins (Table S12). The five most likely candidates for rhicadhesin (sizes range from 16 to 19 kDa) are shown in Table 4. RL4733 was the most abundant protein, with RL3968 (*pal*) (approx. 50% of RL4733) and RL0770, RL1635, and RL4441 (*omp19*) (each approx. 10% of RL4733) (Table S12). None of these has the expected attachment pH profile for the rhicadhesin gene; INSeq classification of RL0770 was NE for attachment at all pHs, while RL4733, *pal*, RL1635, and *omp19* were classified ES/DE under all attachment conditions tested (Table 4). In addition, these latter four genes are also non-NE in both the input library and for growth on TY and other media (23, 25, 26, 53), indicating that they are not specifically involved in root attachment. In conclusion, we have been unable to identify any single gene that encodes rhicadhesin in *R. leguminosarum.*

**TABLE 4:** Potential rhicadhesin-encoding genes determined from INSeq analysis or proteomic screening^*f*^^,^^*g*^

Gene	Description	Predicted protein size (kDa)	INSeq classification			Ca_2_^+^-binding domain^*a*^
			pH 6.5	pH 7.0	pH 7.5	
Proteomic analysis of crude adhesin^*b*^						
RL0770^*c*^	Putative phasin, phasin-2 superfamily	16	NE	NE	NE	Yes
**RL1635**^*d*^	Outer membrane protein	19	DE	DE	DE	No
RL3968 (*pal*)^*d*^	OmpA family peptidoglycan-associated lipoprotein	19	ES	DE	ES	No
**RL4441 (*omp19***)^*d*^	Outer membrane protein Omp19	19	DE	DE	DE	No
**RL4733**^*d*^	Conserved hypothetical protein	17	ES	DE	ES	Yes
INSeq analysis^*e*^						
RL1165	Conserved hypothetical protein	12	NE	NE	DE	Yes
RL1166^*c*^	Putative ribonuclease-L-PSP family protein	14	NE	NE	DE	Yes
RL1339	Conserved hypothetical protein	15	NE	NE	DE	No
RL2095^*c*^	Conserved hypothetical protein	15	NE	NE	DE	Yes
RL2643 (*dksA2*)	Putative DnaK suppressor protein	15	NE	NE	DE	No
RL2858	Conserved hypothetical exported protein	13	NE	NE	DE	No
RL4383^*c*^	Putative AsnC family transcriptional regulator	16	NE	DE	DE	Yes

^*a*^ Ca^2+^-binding activity predicted using the IonCom tool (34).

^*b*^ Candidate genes were identified by LC-MS in a proteomic screen of proteins in the crude adhesin band (Fig. 7A).

^*c*^ Gene contains fewer than six TA sites (four genes).

^*d*^ Required for growth in input library and in other media and conditions (23, 25, 26, 53).

^*e*^ Genes identified as encoding candidate rhicadhesin(s) on the basis of their size (encoding a 12–16 kDa protein) and INseq classification: NE under acid, NE/DE/ES under neutral and DE/ES under alkaline conditions.

^*f*^ INSeq classification abbreviations: ES essential, DE defective, NE neutral.

^*g*^ Bold text shows genes for which mutation was attempted.

## DISCUSSION

While there is a core attachome of 22 genes required under all conditions tested (Table 1), it is clear that bacteria attach initially to plant roots using mechanisms that are heavily dependent on environmental pH as illustrated by the number and variety of genes required under different conditions (Tables S4 to S9). Whereas previous models of primary attachment have largely described a dual glucomannan/rhicadhesin system for primary root attachment under different environmental pH conditions (3–6), our work reveals a total of 115 genes whose products are specifically involved in primary attachment to plant roots, under one or more conditions in the range pH 6.5 to pH 7.5 (Fig. 3; Table S3). It is clear that the bacterial cell surface is absolutely critical for primary attachment, and it is easy to see how alterations in the cell surface could affect the weak, non-specific forces (van der Waals forces, electrostatic charge, and hydrophobic interactions) known to be so important at this stage (reviewed in reference 3). However, at a given pH, different genes are required, presumably reflecting the influence of pH on those initial interactions. Examples of key genes are those encoding either structural components of EPS/membranes or enzymes carrying out key reactions to build or modify those structures. Many and diverse methods of dealing with stress arising from environmental conditions are also involved in successful primary root attachment. We undertook to explore the effects of two regulators identified through INSeq and revealed widespread disruption of expression of numerous genes, with a large proportion of these affecting the bacterial cell surface.

With reference to the dual glucomannan/rhicadhesin primary attachment model, we confirmed the requirement for glucomannan at pH 6.5–7.0 (both by INSeq classification of *gmsA* encoding glucomannan synthetase and the reduced attachment of a mutant strain) and examined the hypothesis that rhicadhesin is a single protein required at alkaline pH for attachment using a combination of INSeq and proteomic approaches. While we confirmed that sonicated cell surface fractions do inhibit attachment at alkaline pH, there were no convincing overlaps between proteins identified by proteomics and the INseq analysis. This leads us to conclude that while this fraction has attachment-inhibiting properties, it did not necessarily contain adhesins. Furthermore, INSeq analysis enabled the identification of seven potential proteins, required for attachment with the necessary pH profile and of the expected gene and protein size (approx. 0.4 kb and 14–19 kDa, respectively) (Table 4). While four of these are predicted to bind Ca^2+^ ions, none were present in the proteomic screen. While there are numerous possibilities for this, the evidence from this study is that there appears to be no single specific rhicadhesin needed for alkaline attachment.

Since *R. leguminosarum* is a nitrogen-fixing bacteria in symbiosis with its legume host pea, we have been able to ascertain how disruption of primary attachment factors can affect downstream events leading to successful symbiosis (Table 3). At neutral pH, approximately half of the genes needed for primary attachment were crucial at later stages—meaning that half the genes were not needed. Symbiosis should perhaps not be regarded as a completely linear process, rather that there are many ways to reach the goal with “marginal gains” affecting bacterial fitness in any given situation.

Despite plasmid-encoded genes making up approx. 35% of the Rlv3841 genome (52), they make up only approx. 17% of those required for primary attachment to roots (19 of 115, Table S3), meaning that there is a bias toward chromosomal genes. However, plasmid genes, particularly of pRL8, are upregulated in the pea rhizosphere within 24 h of inoculation (14), suggesting that plasmid genes may be involved in later stages of, possibly host-specific, secondary attachment and colonization of roots. To investigate this, INSeq secondary attachment/colonization studies over a longer time-scale (>24 h) would be needed.

We coupled INSeq with an existing technique (13), modified for whole root Lux-assays, to examine primary attachment to pea roots, with a standardized 1-h assay, under different pH conditions. We suggest that between the two ways to assess attachment, there is extremely good consensus, with 79% agreement (31/39 experiments), validating the INseq experimental approach. The complete agreement would not be expected as the two experimental approaches differ in the fine details; in INSeq, a mutant library (up to 115,000 mutant strains) is co-inoculated onto roots, while the Lux-based system assesses attachment of a homogeneous population (WT or single mutant strain). These experimental differences can not only lead to apparent discrepancies in results but also highlight the limitations/strengths of the two techniques for assessing bacterial attachment. With evidence that the mutation in RL2969 can be complemented *in trans* by the presence of WT bacteria in the attachment assay (explaining the INSeq classification of RL2969 as NE [Table 2]), this leaves only 13% unexplained differences between the two approaches. We have established that the INSeq classification from a gene (*praR*) containing only four TA sites (located within the central 80% of the coding sequence) disagrees with Lux attachment assay results, which is in line with a suggested limit of six TA sites as a minimum for confident INSeq classification assignment (73, 74). Taking this into account, unexplained differences lie at <10% (2/39 experiments).

In conclusion, we have shown how both specific and generalized changes to the bacterial cell surface and disruption of mechanisms by which bacteria counteract the effects of stress lead to a reduction in primary attachment. This is most likely due to perturbation of van der Waals forces, as well as electrostatic and hydrophobic interactions between the bacteria and roots, which are also influenced by pH. Disruption of primary attachment can influence downstream events and affect symbiosis. This research has initiated a deeper understanding of how environmental pH affects rhizobial attachment to roots, a key interaction between bacteria and plants, prior to bacterial colonization.

## Data Availability

All data needed to evaluate the conclusions in this paper are present in the paper and/or supplemental material. Data from experiments are given in Data sheets S1 to S4. The mass spectrometry proteomics data for the on-bead digestions have been deposited to the ProteomeXchange Consortium via the PRIDE (77) partner repository (https://www.ebi.ac.uk/pride/archive/) with the data set identifier PXD029089. RNASeq data have been uploaded to the NCBI Sequence Read Archive (SRA) database with the accession number PRJNA811156, and INSeq data have been uploaded with the accession number PRJNA1049484 (individual samples are listed in Fig. S3).

## SUPPLEMENTAL MATERIAL

Supplemental figures and tables msystems.00422-24-s0001.docx Fig. S1 to S4 and Tables S1 to S3.

Supplemental data sheets and tables msystems.00422-24-s0002.xlsx Data sheets S1 to S4; Tables S4 to S12.

ASM does not own the copyrights to Supplemental Material that may be linked to, or accessed through, an article. The authors have granted ASM a non-exclusive, world-wide license to publish the Supplemental Material files. Please contact the corresponding author directly for reuse.
