# Supplementary material for: Factors governing attachment of Rhizobium leguminosarum to legume roots at acid, neutral, and alkaline pHs
Source: mSystems. 2024 Aug 21;9(9):e00422-24. doi: 10.1128/msystems.00422-24 (PMC11406972; doi:10.1128/msystems.00422-24)
Supplement: Supplemental figures and tables — Fig. S1 to S4 and Tables S1 to S3. [file msystems.00422-24-s0001.docx]

Supplementary Information

**Factors governing attachment of *Rhizobium leguminosarum* to legume roots**

Jack D. Parsons^1^, Clare R. Cocker^1^, Alison K. East^1^, Rachel M. Wheatley^1^, Vinoy K. Ramachandran^1^, Farnusch Kaschani^2,3^, Markus Kaiser^2^, Philip S. Poole^1^

^1^ Department of Biology, South Parks Road, University of Oxford, Oxford, OX1 3RB, United Kingdom
^2^ Department of Chemical Biology, ZMB, University Duisburg-Essen, Universitätsstr Str. 2, 45117 Essen, Germany

^3^ Analytics Core Facility Essen (ACE), University Duisburg-Essen, ZMB, Universitätsstr. 2, 45117 Essen, Germany

Datasheet S1. INSeq classification for Rlv3841 genes for pea root attachment at pH 6.5, pH 7.0 and pH 7.5 in 1-h assay (>5,000 genes) Excel

Datasheet S2. 280 genes required over pH 6.5-7.5 Excel

Datasheet S3. RNASeq data for strains OPS1907 and OPS1908 Excel

Datasheet S4. Bacterial attachment to pea roots after 1-h incubation Excel

Supplementary Figure S1. Changes in the pH of rooting solution incubated with fine vermiculite over 72 h. Word (attached)

Supplementary Figure S2. Recovery of bacteria from pea roots after 1 h. Word (attached)

Supplementary Figure S3. Library preparation and samples sequenced. Word (attached)

Supplementary Figure S4. Effect of co-inoculation with wild-type bacteria on primary attachment to pea roots of a mutant at different pHs. Word (attached)

Supplementary Table S1. Strains and plasmids. Word (attached)

Supplementary Table S2. Primers and adaptors. Word (attached)

Supplementary Table S3. 115 genes affected in attachment pH 6.5- 7.5. Word (attached)

Supplementary Table S4. Genes required at pH 6.5. Excel

Supplementary Table S5. Genes required at pH 7.0 Excel

Supplementary Table S6. Genes required at pH 7.5 Excel

Supplementary Table S7. Genes required at pH 6.5 and 7.0 Excel

Supplementary Table S8. Genes required at pH 7.0 and 7.5 Excel

Supplementary Table S9. Genes required at pH 6.5 and 7.5 Excel

Supplementary Table S10. Rlv3841 genes with RpoH1 recognition sequence upstream Excel

Supplementary Table S11. Rlv3841 genes with RpoH1/H2 recognition sequences Excel

Supplementary Table S12. Proteins in crude adhesin fraction determined by LC-MS Excel


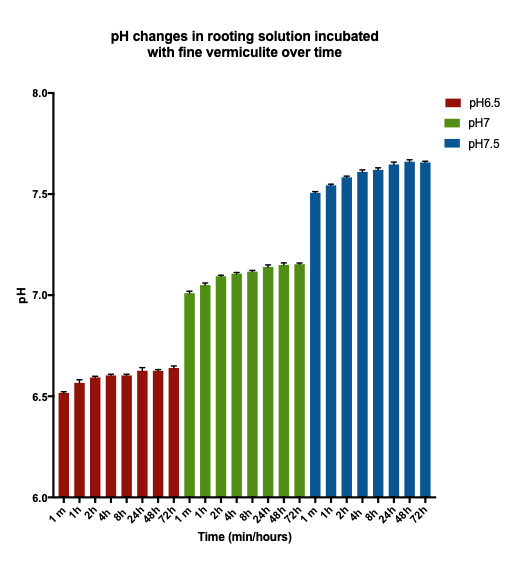


**Supplementary Fig. S1.** **Changes in the pH of rooting solution incubated with fine vermiculite over 72 h.** Initial pH (pH 6.5 (red), pH 7.0 (green) and pH 7.5 (blue)) of rooting solution incubated with fine vermiculite shows little change over a 72-h period. Bars show ±SEM. *n* = 3.


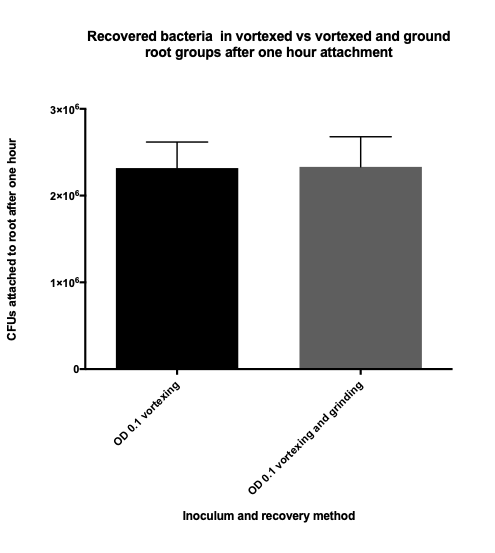


**Supplementary Fig. S2.** **Recovery of bacteria from pea roots after 1 h.** Inoculation was by incubation in 15 ml Rlv3841 (OD_600_ = 0.1). Bacterial attachment was measured in colony forming units (CFUs) released from roots by 1) vortexing alone or 2) vortexing and grinding with a mortar and pestle. *n* = 5. There was no significant difference between the two recovery methods by Student’s t test. Bars show ±SEM.


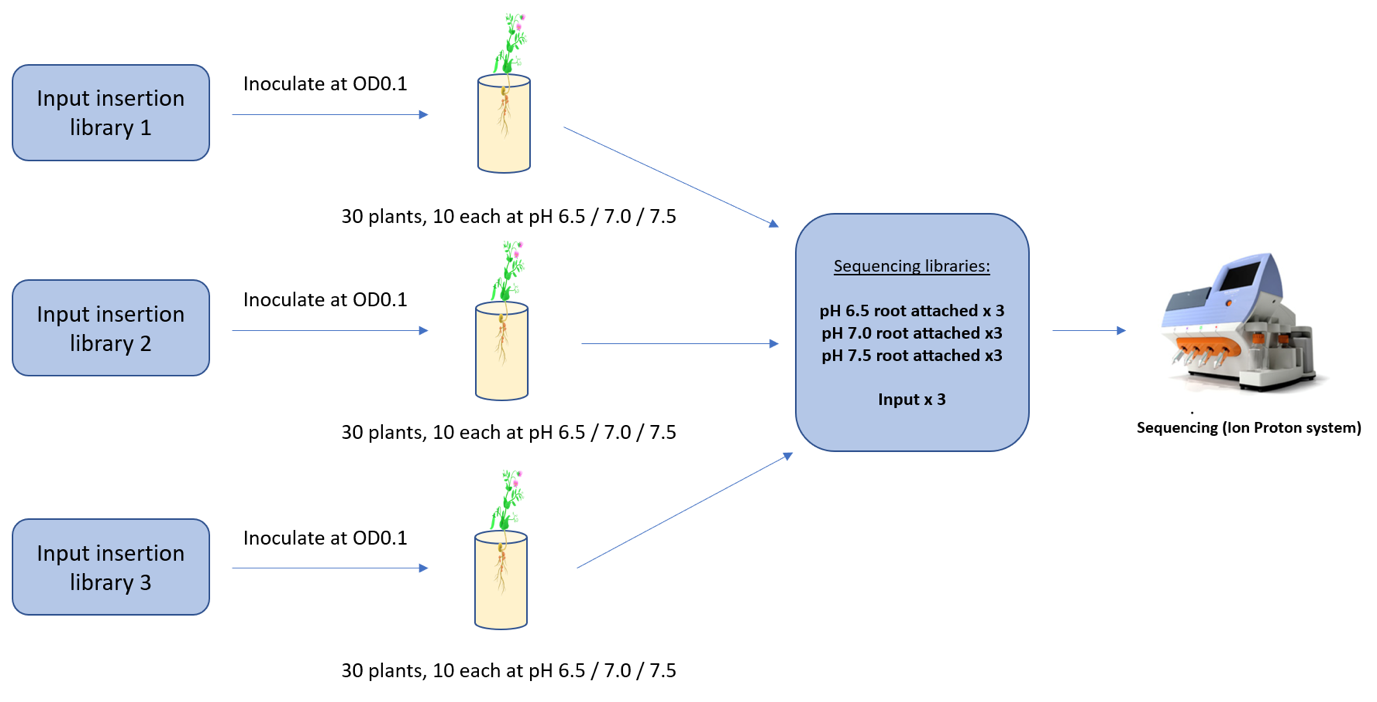


SAMN38698945: Attachment pH6.5_1

SAMN38698946: Attachment pH7.0_1

SAMN38698947: Attachment pH7.5_1

SAMN38698948: Attachment pH6.5_2

SAMN38698949: Attachment pH7.0_2

SAMN38698950: Attachment pH7.5_2

SAMN38698951: Attachment pH6.5_3

SAMN38698952: Attachment pH7.0_3

SAMN38698953: Attachment pH7.5_3

SAMN38698954: Input_1

SAMN38698955: Input_2

SAMN38698956: Input_3

**Supplementary Fig. S3.** **Library preparation and samples sequenced (together with biosample numbers).** 50 mL of input insertion library (1, 2 and 3) was inoculated onto each pea plant root (ten per treatment) at pH 6.5, 7.0 or 7.5 (resulting in three replicates for each pH) and incubated for 1 h before recovery by vortexing. Recovered bacteria for each attachment group were pelleted and grown on TY agar for 12 hr to increase bacterial gDNA concentration (and effectively decrease plant DNA contamination) before DNA preparation. Input library gDNA samples were extracted directly from input inoculum. Each sample (input (1-3) and 3 root-attached replicates at 3 different pHs (a total of 12)) underwent DNA extraction and library preparation before sequencing. Biosample numbers are for NCBI SRA INSeq data submission with the accession number PRJNA1049484.

**Supplementary Fig. S4.** **Effect of co-inoculation with wild-type bacteria on primary attachment to pea roots of a mutant at different pHs.** Comparison of Rlv3841 (WT) and a strain mutated in RL3273, as single inoculum (Mutant) at **A** pH 6.5, where there is no significant difference in root attachment, and at **B** pH 7.0 and **C** pH 7.5 together with 1:1 and 1:100 co-inoculation with unlabeled Rlv3841 (Mutant:WT) using the Lux-based whole root attachment assay. Luminescence (RLU/g of root) shows bacterial attachment after 1 h. *n* ≥ 9. All data points are shown with the box indicating the interquartile range and the median shown. Maximum and minimum values are indicted by the whiskers. ***= *p* < 0.001, *= *p <* 0.05 using Student’s t test*.* An unpaired t-test was used to compare groups.

**Supplementary Table S1. Strains and plasmids used in this work.**

| Strain/Plasmid | Description | Reference |
| --- | --- | --- |
|  |  |  |
| Rlv3841 | ﻿*R. leguminosarum* bv. *viciae*, wild-type, streptomycin-resistant derivative of Rlv300 | [1] |
| *E. coli* SM10λpir | ﻿*thi-1 thr leu tonA lacY supE recA*::RP4-2-Tc::Mu KanR λpir | [2] |
| A963 | *praR* mutant of Rlv3841, RL0390::Tn*5* | [3] |
| A1045 | *gmsA* mutant of Rlv3841, RL1661::Tn*5* | [4] |
| A1430 | ﻿*praR* *rapA2/C* mutant of Rlv300, *praR::*Tn*5 rapA2*ΩSpec *rapC*::Tn*5*ΩApra | [5] |
| LMB310 | *pssA* mutant of Rlv3841, RL3752::::TnΩSpec | [6] |
| LMB349 | Mutant of Rlv3841, RL3273::pK19 | [7] |
| OPS2051 | Mutant of Rlv3841, pRL110543::pK19 | This work |
| OPS2052 | Mutant of Rlv3841, pRL110071::pK19 | This work |
| OPS2053 | Mutant of Rlv3841, pRL100053::pK19 | This work |
| OPS2054 | Mutant of Rlv3841, RL0109::pK19 | This work |
| OPS1782 | Mutant of Rlv3841, RL4382::pK19 | This work |
| OPS1783 | Mutant of Rlv3841, RL2969::pK19 | This work |
| OPS1907 | Mutant of Rlv3841, RL3453::pK19 |  |
| OPS1908 | Mutant of Rlv3841, RL4145::pK19 | This work |
| RU4062 | *nifH* mutant of Rlv3841, pRL100162::pK19 | [8] |
| Plasmids |  |  |
| pIJ11282 | ﻿Broad host-range plasmid pIJ11268 with *luxCDABE* expressed from constitutive nptII promoter, TetR, AmpR | [5] |
| pOPS0478 | 284 bp fragment of pRL100053 PCR-amplified with oxp1751/1752 and cloned into *Hin*dIII-digested pK19mob, KanR | This work |
| pOPS0479 | 668 bp fragment of pRL110071 PCR-amplified with oxp1753/1754 and cloned into *Hin*dIII-digested pK19mob, KanR | This work |
| pOPS0480 | 325 bp fragment of pRL110543 PCR-amplified with oxp1755/1756 and cloned into *Hin*dIII-digested pK19mob, KanR | This work |
| pOPS0483 | 341 bp fragment of RL0109 PCR-amplified with oxp1785/1786 and cloned into *Hin*dIII-digested pK19mob, KanR | This work |
| pOPS0487 | Fragment of RL2969 PCR-amplified with oxp1767/oxp1768 and cloned into *Hin*dIII-digested pK19mob, KanR | This work |
| pOPS0489 | 1035 bp fragment of RL4382 PCR-amplified with oxp1777/1778 and cloned into *Hin*dIII-digested pK19mob, KanR | This work |
| pOPS1294 | Fragment of RL3453 PCR-amplified with oxp3235/oxp3236 and cloned into *Hin*dIII-digested pK19mob, KanR | This work |
| pOPS1295 | Fragment of RL4145 PCR-amplified with oxp3231/oxp3232 and cloned into *Hin*dIII-digested pK19mob, KanR | This work |
| pSAM_Rl | ﻿pSAM_Km with *B. thetatiotamicron* RpoD promoter replaced with Rlv3841 RpoD promoter region, AmpR, KanR | [2] |

**Supplementary Table S2. Primers used in this work.**

| **Primer** | **Description** | **Sequence (5’-3’)** |
| --- | --- | --- |
|  |  |  |
| oxp0434 | BioSamA primer for INSeq linear PCR | CGGTTCGCTTGCTGTCCATAAAACC |
| oxp0435 | M12 top primer for INSeq | CTGTCCGTTCCGACTACCCTCCCGAC |
| oxp0436 | M12 bottom primer for INSeq | GTCGGGAGGGTAGTCGGAACGGACAG |
| oxp0437 | Sequencing adapter primer 1 for INSeq | AGATCGGAAGAGCGTCGTGTAGGGAA |
| oxp0438 | Sequencing adapter primer 2 for INSeq | TTCCCTACACGACGCTCTTCCGATCTNN |
| oxp0439 | INSeq forward barcode primer 1 | CCATCTCATCCCTGCGTGTCTCCGACTCAGCTAAGGTAACGATATAAAACCGCCCAGTCTACTCGAGGG |
| oxp0440 | INSeq forward barcode primer 2 | CCATCTCATCCCTGCGTGTCTCCGACTCAGTAAGGAGAACGATATAAAACCGCCCAGTCTACTCGAGGG |
| oxp0441 | INSeq forward barcode primer 3 | CCATCTCATCCCTGCGTGTCTCCGACTCAGAAGAGGATTCGATATAAAACCGCCCAGTCTACTCGAGGG |
| oxp0442 | INSeq forward barcode primer 4 | CCATCTCATCCCTGCGTGTCTCCGACTCAGTACCAAGATCGATATAAAACCGCCCAGTCTACTCGAGGG |
| oxp0443 | INSeq forward barcode primer 5 | CCATCTCATCCCTGCGTGTCTCCGACTCAGCAGAAGGAACGATATAAAACCGCCCAGTCTACTCGAGGG |
| oxp0444 | INSeq forward barcode primer 6 | CCATCTCATCCCTGCGTGTCTCCGACTCAGCTGCAAGTTCGATATAAAACCGCCCAGTCTACTCGAGGG |
| oxp0445 | INSeq forward barcode primer 7 | CCATCTCATCCCTGCGTGTCTCCGACTCAGTTCGTGATTCGATATAAAACCGCCCAGTCTACTCGAGGG |
| oxp0446 | INSeq forward barcode primer 8 | CCATCTCATCCCTGCGTGTCTCCGACTCAGTTCCGATAACGATATAAAACCGCCCAGTCTACTCGAGGG |
| oxp0447 | INSeq forward barcode primer 9 | CCATCTCATCCCTGCGTGTCTCCGACTCAGTGAGCGGAACGATATAAAACCGCCCAGTCTACTCGAGGG |
| oxp0448 | INSeq forward barcode primer 10 | CCATCTCATCCCTGCGTGTCTCCGACTCAGCTGACCGAACGATATAAAACCGCCCAGTCTACTCGAGGG |
| oxp0449 | INSeq forward barcode primer 11 | CCATCTCATCCCTGCGTGTCTCCGACTCAGTCCTCGAATCGATATAAAACCGCCCAGTCTACTCGAGGG |
| oxp0450 | INSeq forward barcode primer 12 | CCATCTCATCCCTGCGTGTCTCCGACTCAGTAGGTGGTTCGATATAAAACCGCCCAGTCTACTCGAGGG |
| oxp0451 | Reverse barcode primer for INSeq | CCTCTCTATGGGCAGTCGGTGATTTCCCTACACGACGCTCTTCCGATCT |
| oxp1751 | pRL100053 PCR for cloning, forward | TGATTACGCCAAGCTATGGTTGCCATCAAGC |
| oxp1752 | pRL100053 PCR for cloning, reverse | GCAGGCATGCAAGCTTCTTTGAAGCGATCACGGGC |
| oxp1753 | pRL110071 PCR for cloning, forward | TGATTACGCCAAGCTTGCGGGAAGGGGCGT |
| oxp1754 | pRL110071 PCR for cloning, reverse | GCAGGCATGCAAGCTCGAAATTCGCTGCGGAAAAC |
| oxp1755 | pRL110543 PCR for cloning, forward | TGATTACGCCAAGCTCTGCCGAAGCCAAGG |
| oxp1756 | pRL110543 PCR for cloning, reverse | GCAGGCATGCAAGCTCGTTCGGCCTTTCGGG |
| oxp1767 | RL2969 PCR for cloning, forward | TGATTACGCCAAGCTGTCCAGTCGGCGCCG |
| oxp1768 | RL2969 PCR for cloning, reverse | GCAGGCATGCAAGCTAGCGGCTGTAGCAATAGTCG |
| oxp1777 | ﻿RL4382 PCR for cloning, forward | TGATTACGCCAAGCTGATCTCCGGAACCATTGCC |
| oxp1778 | RL4382 PCR for cloning, reverse | GCAGGCATGCAAGCTGGTGCGGCGGGTGTG |
| oxp1785 | RL0109 PCR for cloning, forward | TGATTACGCCAAGCTACCGGCAAGAAGTTTTATGATCT |
| oxp1786 | RL0109 PCR for cloning, reverse | GCAGGCATGCAAGCTATGATGTCGCTCATGTCGTCATC |
| oxp2052 | RL2969::pK19 mapping primer, forward | TATCTCCCCCGCCGCGTTAT |
| oxp2053 | RL2969::pK19 mapping primer, reverse | GCGAGGCTTGCTCCGAT |
| oxp2058 | RL4382::pK19 mapping primer, forward | TTCGCTCGATTTTACCAAGC |
| oxp2059 | RL4382::pK19 mapping primer, reverse | AGATTGCGGACCGACGT |
| oxp2062 | pRL100053::pK19 mapping primer, forward | GAAGGAGATGGACACTGCAC |
| oxp2063 | pRL100053::pK19 mapping primer, reverse | CACCAGGGATGAAAGCTTGA |
| oxp2064 | pRL110071::pK19 mapping primer, forward | AGTTCGATTGACAGGCTCTC |
| oxp2065 | pRL110071::pK19 mapping primer, reverse | GTGGAATTCTGCTGCTTCG |
| oxp2066 | pRL110543::pK19 mapping primer, forward | CGAAGTCAAACAGTCAGGAA |
| oxp2067 | pRL110543::pK19 mapping primer, reverse | CGCTGCCTGTCCTGAC |
| oxp2068 | RL0109::pK19mapping primer, forward | GGCTATTCACACGAGGCTC |
| oxp2069 | RL0109::pK19 mapping primer, reverse | TTTTTCCTCGCCGGGC |
| oxp3231 | RL4145 PCR for cloning, forward | TGATTACGCCAAGCTATTGACATAGCCCTGGTAGC |
| oxp3232 | RL4145 PCR for cloning, reverse | GCAGGCATGCAAGCTCGGGAGAAGATCAAGGAACA |
| oxp3233 | RL4145::pK19mapping primer, forward | CTCATCGTTCAGCACATCAG |
| oxp3234 | RL4145::pK19 mapping primer, reverse | CCTGAGCAATGTCATATCGC |
| oxp3235 | RL3453 PCR for cloning, forward | TGATTACGCCAAGCTGAAATCGGATTCCAGCTTCG |
| oxp3236 | RL3453 PCR for cloning, reverse | GCAGGCATGCAAGCTTGCCTCGAACACACATATCT |
| oxp3237 | RL3453::pK19 mapping primer, forward | GAAAGAAGGTACCCAGACGA |
| oxp3238 | RL3453::pK19mapping primer, reverse | CAGATATTTCCGACGATGCG |

**Supplementary Table S3. One hundred and fifteen genes required specifically for root attachment at pH 6.5 - pH 7.5 (their distribution is shown in Fig. 3).**

| **No.** | **Gene** | **Name** | **Description** | | **Required** |
| --- | --- | --- | --- | --- | --- |
| 1 | pRL100053^1^ |  | Putative transmembrane domain containing protein, helix-turn-helix 37 domain. | | pH 6.5, pH 7.0, pH 7.5 |
| 2 | pRL100112 |  | Putative dehalogenase-hydrolase (HAD), member of a large superfamily of phosphohydrolases. HADs display activity against various phosphorylated metabolites [9]. A mutant in a *Xanthomonas axonopodis* pv. *citri* HAD is defective in biofilm formation [10]. | | pH 7.0 |
| 3 | pRL100162A |  | Hypothetical protein. Unknown localisation. | | pH 6.5, pH 7.5 |
| 4 | pRL100163 |  | Uncharacterised cytoplasmic protein, asparagine synthetase family domain. | | pH 6.5, pH 7.5 |
| 5 | pRL100174 |  | Hypothetical protein, no known conserved domains. Unknown localisation. | | pH 6.5, pH 7.0, pH 7.5 |
| 6 | pRL100177 |  | Putative homologue of eukaryotic tubulin. Unknown localisation. | | pH 6.5 |
| 7 | pRL100220 |  | Uncharacterised protein. | | pH 6.5, pH 7.0 |
| 8 | pRL100242 |  | Uncharacterised cytoplasmic protein. | | pH 6.5, pH 7.0 |
| 9 | pRL100274 | *fucA* | Putative α-L-fucosidase. Cleaves fucosidic bonds in glycans (particularly in peptidoglycan structures) [11]; likely involved in remodelling of the cell surface. Fucose-rich EPS has been documented in *R. sullae* [12]. | | pH 6.5 |
| 10 | pRL100470 |  | Uncharacterised protein. Unknown localisation. | | pH 6.5, pH 7.5 |
| 11 | pRL110043 |  | MFS family transporter protein. 99% similarity to arabinose efflux permease in *R. leguminosarum* biovar *trifolii* WSM597. EPS arabinose content regulates cell aggregation in *Azospirillum* [13]. | | pH 6.5, pH 7.0 |
| 12 | pRL110045^2^ |  | Uncharacterised protein. Unknown localisation. | | pH 6.5, pH 7.5 |
| 13 | pRL110046 |  | Putative FNR/CRP family transcriptional regulator. Cytoplasmic protein. | | pH 6.5, pH 7.5 |
| 14 | pRL110283 |  | Putative ArsR family transcriptional regulator. Cytoplasmic protein. | | pH 6.5 |
| 15 | pRL120021 |  | Uncharacterised protein. Unknown localisation. | | pH 6.5 |
| 16 | pRL120322 | *fhuA2* | Outer membrane siderophore receptor FhuA2. | | pH 7.5 |
| 17 | pRL120475 | *impA* | Inner membrane protein ImpA. Mutation of *impA* causes outer membrane disruption in *Actinobacillus actinomycetemcomitans* [14]. Expressed from the pathogenic injection type VI secretion system operon in *V. cholerae* [309]. Nicolsamide reduces *Xanthomonas oryzae* leaf blight disease in rice by downregulating xanthan, EPS and *impA* expression [15]. | | pH 6.5 |
| 18 | pRL120518 |  | TetR family transcriptional regulator. | | pH 7.0 |
| 19 | pRL120795 |  | Uncharacterised protein. Unknown localisation. | | pH 7.5 |
| 20 | RL0032^2^ | *npr* | Components of the phosphenolpyruvate phosphotransferase (PTS) system regulate lifestyle switches [6]. Phospho-relay between Npr and ManX is believed to regulate carbon metabolism [16]. Npr and ManX are co-localised with the ChvI operon [17] and globally regulate ATP-dependent ABC transporter activity in a post-translational fashion [6, 18]. Mutation of PTS system components causes dry colony morphology due to reduced EPS secretion [18]. | | pH 6.5 |
| 21 | RL0033 | *manX* | Components of the phosphenolpyruvate phosphotransferase (PTS) system regulate lifestyle switches [6]. Phospho-relay between Npr and ManX is believed to regulate carbon metabolism [16]. Npr and ManX are co-localised with the ChvI operon [17] and globally regulate ATP-dependent ABC transporter activity in a post-translational fashion [6, 18]. Mutation of PTS system components causes dry colony morphology due to reduced EPS secretion [18]. | | pH 6.5 |
| 22 | RL0052 |  | Uncharacterised protein. Unknown localisation. | | pH 7.0 |
| 23 | RL0141 | *cycM* | Membrane-bound cytochrome c CycM. | | pH 7.0, pH 7.5 |
| 24 | RL0395 | *miaB* | tRNA-2-methylthio-N(6)-dimethylallyladenosine synthase. | | pH 7.5 |
| 25 | RL0398 |  | Putative N-acetyltransferase (NAT). STRING database indicates functional interaction with RL0399 (putative endopeptidase, interaction score 0.91) and MurE (peptidoglycan biosynthesis factor, interaction score 0.71) – possibly related to alterations in peptidoglycan structure. | | pH 6.5 |
| 26 | RL0401 |  | Putative universal stress protein, UspA family. | | pH 7.5 |
| 27 | RL0551 | *hslO* | Putative Hsp33-like chaperonin. Redox regulated molecular chaperone protecting thermally unfolding and oxidised proteins from aggregation – defence against oxidative stress [19, 20]. May assist in coping with a diverse rhizosphere environment. | | pH 6.5, pH 7.0, pH 7.5 |
| 28 | RL0561 |  | AraC family transcriptional regulator. | | pH 7.5 |
| 29 | RL0614 |  | Unknown function. Cytoplasmic protein. | | pH 6.5 |
| 30 | RL0617 |  | Putative dTTP/UTP pyrophosphatase. | | pH 7.0, pH 7.5 |
| 31 | RL0726 |  | Conserved hypothetical exported protein, transglycosylase Slt domain. Degrades peptidoglycan via β 1-4 glycosidic bond cleavage. Linked to biofilm formation in *S. enterica [21]*, *E. coli* [22] and *Acinetobacter baumannii* [23]. Lytic transglycosylases participate extensively in cell wall remodelling, recycling of peptidoglycan and space-making for insertion of cell-envelope spanning structures [24]. A lytic transglycosylase (RL4716) was characterised in Rlv3841 as required for cell envelope function and biofilm formation [25]. | | pH 6.5 |
| 32 | RL0876 |  | Conserved hypothetical cytoplasmic protein, no known conserved domains. | | pH 6.5, pH 7.0, pH 7.5 |
| 33 | RL1013 |  | Uncharacterised protein. Unknown localisation. 17kDa Anti 2 motif common in membrane proteins, including surface antigens in *Rickettsia* [26]. | | pH 7.0 |
| 34 | RL1026 | *ctaE* | Putative cytochrome c oxidase polypeptide III. Biofilm formation is promoted by some cytochrome c oxidases under anoxic conditions in *P. aeruginosa*, possibly due to nitric oxide (NO) accumulation [27]. | | pH 6.5 |
| 35 | RL1052 |  | Uncharacterised protein. Unknown localisation. | | pH 7.0 |
| 36 | RL1106^2^ | *pspA* | Putative PspA family regulator, phage shock protein A. Involved in antibiotic resistance and biofilm formation in *E. coli* and pathogenesis in *S. typhimurium* [28, 29]. | | pH 7.0 |
| 37 | RL1164 |  | Uncharacterised protein, unknown localisation. PepSY domains likely to have a protease inhibitory function and may be cell wall-associated [30]. Biofilm metalloprotease 1 (BmpI) from *Pseudoalteromonas* contains a PepSY domain required for biofilm formation [31]*.* | | pH 7.5 |
| 38 | RL1165 |  | Uncharacterised protein, 93% identity to gene RLV_3555 from *R. leguminosarum* biovar *viciae*, PepSY domains are likely to have a protease inhibitory function and may be cell wall-associated [30]. Biofilm metalloprotease 1 (BmpI) from *Pseudoalteromonas* contains a PepSY domain required for biofilm formation [31]*.* | | pH 7.5 |
| 39 | RL1338^2^ | *pmtA* | Putative phosphatidylethanolamine (PE) N-methyltransferase PmtA. Significantly upregulated in a *praR* mutant of Rlv3841, suggesting attachment role [5]. Phosphatidylcholine is found in the membranes of Rhizobiaceae and is synthesised from PE by PmtA. *pmtA* mutation in *Bradyrhizobium japonicum* disrupts symbiosis formation [32]. | | pH 7.5 |
| 40 | RL1339 |  | Uncharacterised protein. | | pH 7.5 |
| 41 | RL1371^2^ |  | Putative transmembrane protein. | | pH 7.0 |
| 42 | RL1381 |  | Uncharacterised protein. Unknown localisation. | | pH 6.5, pH 7.0, pH 7.5 |
| 43 | RL1478 | *amn* | AMP nucleosidase; catalyses hydrolysis of AMP to form adenine and ribose 5-phosphate. Changes in AMP levels allow rapid adjustments to changing metabolic conditions [33]. | | pH 6.5, pH 7.0, pH 7.5 |
| 44 | RL1504 |  | Uncharacterised cytoplasmic protein, NYN domain. Possibly novel RNAse with regulatory role [34]. | | pH 7.0, pH 7.5 |
| 45 | RL1505^2^ | *rpoZ* | DNA-directed RNA polymerase subunit omega, RpoZ. The smallest RNA polymerase subunit. However, additional roles have been identified: deletion of RpoZ impairs biofilm formation and sliding motility in *M. smegmatis* [35] and biofilm formation in *E. coli* [36] and *S. aureus* [37] through transcriptional changes. In *S. aureus*, *rpoZ* deletion impairs production of two tributyrin lipases. Loss of these lipases impairs biofilm formation; this may partly explain the *rpoZ* mutant phenotype [38]. | | pH 7.5 |
| 46 | RL1552 | *rpiI* | 50S ribosomal protein L9 RpiI. | | pH 7.5 |
| 47 | RL1600 | *ppx* | Putative exopolyphosphatase Ppx. | | pH 6.5, pH 7.0 |
| 48 | RL1661^1^ | *gmsA* | Glucomannan biosynthesis protein GmsA. Characterised factor important for polar primary root attachment at acidic but not alkaline pH [4]. | | pH 6.5, pH 7.0 |
| 49 | RL1805 |  | Putative transmembrane protein. | | pH 6.5, pH 7.5 |
| 50 | RL1806 | *degQ* | Periplasmic serine endoprotease DegQ. In *B. subtilis* DegQ stimulates phosphor-transfer to a transcriptional regulator affecting biofilm formation, promoting transition from a motile to sessile attached state [39]*.* | | pH 6.5, pH 7.5 |
| 51 | RL2044 | *scpA* | Segregation and condensation protein A, participates in chromosomal division during cell partition. | | pH 7.0 |
| 52 | RL2080 |  | Putative acetyltransferase. | | pH 7.5 |
| 53 | RL2081^2^ |  | Putative transmembrane protein. | | pH 7.5 |
| 54 | RL2083 |  | Putative acetyltransferase. | | pH 7.5 |
| 55 | RL2094 |  | Putative polyhydroxybutarate (PHB) synthase. | | pH 7.5 |
| 56 | RL2095^2^ |  | Uncharacterised protein. Unknown localisation. | | pH 7.5 |
| 57 | RL2098 |  | Putative transmembrane protein. | | pH 6.5, pH 7.5 |
| 58 | RL2211 |  | Uncharacterised protein. Phasin 2 domain commonly found in phasin proteins which stabilise PHB storage granules [40]. Unknown localisation. | | pH 6.5 |
| 59 | RL2212 | *clpS* | ATP-dependent Clp protease adaptor protein ClpS. ClpS modifies ClpA substrate specificity [364]. *P. aeruginosa clpS* mutant showed 70% reduction biofilm formation and significant impairment in attachment to abiotic surfaces after 1 h [41]. | | pH 7.0, pH 7.5 |
| 60 | RL2227 |  | Zinc metalloprotease. STRING database indicates functional interaction with CdsA (RL2266, phospholipid metabolism, score 0.92) and BamA (RL2228, outer membrane assembly factor, score 0.87) suggesting a role in membrane lipid processing. | | pH 6.5, pH 7.0 |
| 61 | RL2284 | *hfq* | RNA-binding protein Hfq. Global post transcriptional regulator. Loss of Hfq in *S. meliloti* delays nodulation and reduces competitiveness for attachment to alfalfa roots [42]. | | pH 6.5, pH 7.0 |
| 62 | RL2285 | *hflX* | GTPase HflX. Heat-shock induced ribosome splitting factor; rescues translationally stalled ribosomes under heat shock [43]. | | pH 6.5 |
| 63 | RL2303 | *ccdA* | Putative cytochrome c-type biogenesis protein, DsbD transmembrane domain, disulphide bond formation and redox condition maintenance [44]. | | pH 6.5 |
| 64 | RL2316 |  | Putative guanylate cyclase. Catalyses formation of cyclic di-GMP (c-di-GMP), key second messenger in biofilm formation / motile to sessile lifestyle switch [45]. NO can stimulate biofilm formation by regulating c-di-GMP levels in *Shewanella oneidensis* [46]. | | pH 6.5 |
| 65 | RL2394 | *nnrE* | Multifunctional fusion protein NnrE. Repairs epimers of NAD(P)HX, a damaged form of NAD(P)H. | | pH 6.5 |
| 66 | RL2400^2^ |  | Putative MarC family transmembrane protein, function unknown. Cytoplasmic membrane protein. | | pH 6.5, pH 7.0, pH 7.5 |
| 67 | RL2477 | *dacF* | Putative penicillin-binding protein DacF. Functions as a D-alanyl-D-alanine carboxypeptidase in *E. coli* and is thought to be involved in cell wall synthesis and modifications [47]. | | pH 6.5, pH 7.5 |
| 68 | RL2489A^2^ |  | 100% identity to transglycosylase associated protein Rleg_2013 from *R. leguminosarum* biovar *trifolii* WSM1325. Transglycosylation is a key step in bacterial peptidoglycan synthesis, catalysing glycan chain polymerisation [48]. | | pH 7.5 |
| 69 | RL2491^2^ |  | Conserved hypothetical exported protein. | | pH 7.5 |
| 70 | RL2513 | *tpiA* | Putative triosephosphate isomerase. Upregulated in *Staphylococcus aureus* biofilm, possibly due to oxygen limitation [49]. Glycolytic enzymes play additional roles when localised on the cell surface (e.g., α-enolase plasminogen binding in streptococci [50] and GAPDH transferrin binding activity in *S. aureus*) [51]. Surface localised glycolytic enzymes are multifaceted and can be involved in substrate binding. TpiA has been shown to be surface localised and have a direct role in attachment to host cells in *Mycoplasma gallisepticum* [52]. | | pH 6.5, pH 7.0, pH 7.5 |
| 71 | RL2520 |  | Putative transmembrane protein, ABC transporter permease. | | pH 6.5, pH 7.0 |
| 72 | RL2564^2^ |  | Hypothetical exported protein. Unknown localisation. | | pH 6.5 |
| 73 | RL2587 | *anmK* | Anhydro-N-acetylmuramic acid kinase AnmK. Catalyses the phosphorylation of 1,6-anhydro-N-acetylmuramic acid (anhMurNAc), cleaving the 1,6 anhydro ring and generating MurNAc-6-P. Required for cell wall recycling [53, 54]. | | pH 7.0 |
| 74 | RL2588 | *tyrS* | Tyrosine-tRNA ligase TyrS. Catalyses attachment of tyrosine to tRNA. | | pH 7.0 |
| 75 | RL2595 |  | Putative MutT/nudix family protein. Mutagenesis of nudix proteins in *P. syringae* str DC3000 and *P. aeruginosa* display defects in motility and biofilm formation [55]. | | pH 6.5 |
| 76 | RL2637 | *recA* | RecA is needed for DNA repair and the SOS response. The major activity of RecA in DNA metabolism is the promotion of DNA strand exchange [56]. Requirement at all symbiosis stages suggest RecA may assist in coping with a diverse rhizosphere environment. However, *recA* disruption has been shown to reduce adherence and colonization of host cells by *Vibrio cholerae*, although the mechanism underlying this remains unknown [57]. | | pH 6.5, pH 7.0, pH 7.5 |
| 77 | RL2642^2^ |  | Uncharacterised protein. | | pH 7.0, pH 7.5 |
| 78 | RL2643 | *dksA2* | Putative DnaK suppressor protein DksA. Cytoplasmic protein. | | pH 7.0, pH 7.5 |
| 79 | RL2644 | *sixA* | Phosphohistidine phosphatase SixA, conserved. SixA is the only known bacterial phosphohistidine phosphatase, and dephosphorylates Npr in *E. coli* [58]. Implicated in biofilm formation in *E. coli* [59]. | | pH 7.5 |
| 80 | RL2694 | *gor* | Glutathione reductase Gor. | | pH 7.0 |
| 81 | RL2695^2^ |  | Hypothetical protein. Unknown localisation. | | pH 7.0 |
| 82 | RL2777 |  | Uncharacterised cytoplasmic protein. | | pH 7.5 |
| 83 | RL2778^2^ |  | Putative exopolysaccharide biosynthesis protein. | | pH 7.5 |
| 84 | RL2780^2^ | *dgkA* | Diacylglycerol kinase DgkA. In *E. coli,* DgkA mutants are defective in biofilm formation [60], and DgkA function has been linked to phospholipid recycling and LPS modifications [61]. In *B. subtillis* it is important for lipoteichoic acid synthesis [62]. | | pH 6.5 |
| 85 | RL2857 |  | LysR family transcriptional regulator. | | pH 7.5 |
| 86 | RL2858 |  | Conserved hypothetical exported protein, coiled-coil domain. | | pH 7.5 |
| 87 | RL3179 |  | Putative cobalamin (vitamin B12) synthesis protein, CobW domain, may also be involved in peptidoglycan amidation. | | pH 6.5 |
| 88 | RL3226 | *ahpD* | Alkyl hydroperoxide reductase AhpD. | | pH 7.0 |
| 89 | RL3253 | *hflC* | Putative transmembrane serine protease. Functional HflC and HflK (RL3254) modulate HflB activity. HflB, an AAA metalloprotease, is involved in membrane protein regulation, LPS biosynthesis and biofilm formation in *E. coli*, *B. subtilis* and others (where it is often called FtsH) [63, 64] indicating a role in membrane regulation and biofilm formation [65-67]. *hflB* (RL3965) is ES/DE under all conditions, including input library. | | pH 7.5 |
| 90 | RL3254 | *hflK* | Putative transmembrane serine protease. Functional HflC (RL3253) and HflK modulate HflB activity. HflB, an AAA metalloprotease, is involved in membrane protein regulation, LPS biosynthesis and biofilm formation in *E. coli*, *B. subtilis* and others (where it is often called FtsH) [63, 64] indicating a role in membrane regulation and biofilm formation [65-67]. *hflB* (RL3965) is ES/DE under all conditions, including input library. | | pH 7.5 |
| 91 | RL3277 |  | Putative transmembrane protein. | | pH 6.5, pH 7.0 |
| 92 | RL3322 | *pfp* | Putative pyrophosphate-fructose 6-phosphate 1-phosphotransferase. Catalyses the first committed step in glycolysis, the phosphorylation of D-fructose-6-phosphate [68]. Like RL2513, could have multifaceted role. | | pH 6.5, pH 7.0, pH 7.5 |
| 93 | RL3752^1^ | *pssA* | Glycosyl transferase involved in EPS biosynthesis [69, 70]. Mutants are deficient in EPS production and form biofilms slowly compared to Rlv3841, do not attach to root hairs and biofilms are flat and unstructured [58]. | | pH 6.5, pH 7.0, pH 7.5 |
| 94 | RL3766 | *rpoH1* | Putative RNA polymerase sigma-32-factor, heat shock. Involved in the regulation of expression of heat shock genes and stress response; may also confer pH change and osmotic stress tolerance [71]. May assist in coping with a diverse rhizosphere environment. | | pH 6.5, pH 7.0, pH 7.5 |
| 95 | RL3987^2^ |  | Uncharacterised, SpoVT-AbrB domain. Acts as antitoxin of toxin-antitoxin pairings. | | pH 6.5, pH 7.0, pH 7.5 |
| 96 | RL3988 |  | Uncharacterised, PINc domain. PIN domains function as single-stranded RNA nucleases [72]. In prokaryotes they are usually the toxin of toxin-antitoxin operons, helping free-living prokaryotes cope with nutritional stress [73]. | | pH 6.5, pH 7.0, pH 7.5 |
| 97 | RL3989 |  | Holliday junction ATP-dependent DNA helicases RuvA. DNA damage repair mechanism. May be required for osmotic shock responses [74, 75]. | | pH 6.5, pH 7.0, pH 7.5 |
| 98 | RL3990 |  | Holliday junction ATP-dependent DNA helicases RuvB. DNA damage repair mechanism. May be required for osmotic shock responses [74, 75]. | | pH 6.5, pH 7.0, pH 7.5 |
| 99 | RL4018 |  | Putative ATP-binding component of ABC transporter. 96% identity to Lipid A ABC exporter from *R. leguminosarum* biovar *trifolii* WSM2304 Rleg2_3249. Mutants with reduced lipid A show a delay in nodulation onset and impaired bacteroid shape [76]. Further, defects in lipid A production reduce surface attachment and motility [77]. | | pH 7.5 |
| 100 | RL4062 |  | Putative amidohydrolase. | | pH 7.5 |
| 101 | RL4063^2^ |  | Uncharacterised protein. Unknown localisation. | | pH 7.5 |
| 102 | RL4065^2^ |  | Conserved hypothetical cytoplasmic protein, no conserved domains. | | pH 6.5, pH 7.0, pH 7.5 |
| 103 | RL4075 |  | Putative 5’-nucleotidase. | | pH 7.5 |
| 104 | RL4083 |  | Uncharacterised protein, SGHN family esterase domain. Unknown localisation. | | pH 7.0, pH 7.5 |
| 105 | RL4145^1^ | *pckR* | LacI family transcriptional regulator (repressor). Cytoplasmic protein. | | pH 6.5, pH 7.0, pH 7.5 |
| 106 | RL4309 |  | Putative transmembrane protein. 94% identity to *R. hidalgonense* DedA family protein (CO674_30990). DedA proteins appear to function in membrane homeostasis; mutants show altered membrane lipid composition in multiple bacterial species [78]. | | pH 6.5, pH 7.0 |
| 107 | RL4335 |  | Uncharacterised protein. Unknown localisation. | | pH 6.5, pH 7.0 |
| 108 | RL4362^2^ |  | Putative cobalamin (vitamin B12) synthesis protein, CobW domain [79]. Required by *S. meliloti* for symbiosis with *M. sativa* [80]. Only one cobalamin dependent enzyme (*nrdJ*, *S. meliloti* cobalamin dependent ribonucleotide reductase, RNR) affects symbiosis. Removal of *ndrJ* impairs symbiosis; rhizobia are lysed in the plant cytoplasm [81]. Loss of RL4362 may reduce fitness for competitive primary attachment. Note that cobalamin synthesis genes may be misclassified due to high homology with glutamine amidotransferases, which are involved in peptidoglycan amidation [82]. | | pH 6.5, pH 7.0, pH 7.5 |
| 109 | RL4363 | *dacC* | Putative penicillin binding protein, peptidase S11 domain. In *E. coli* *dacC* processes sugar-peptide cell wall precursors; involved in peptidoglycan biosynthesis [83]. | | pH 6.5, pH 7.0, pH 7.5 |
| 110 | RL4381 |  | Putative POTRA domain transporter. | | pH 6.5, pH 7.0, pH 7.5 |
| 111 | RL4382^1^ |  | Filamentous hemagglutinin adhesin. | | pH 6.5, pH 7.0, pH 7.5 |
| 112 | RL4383 |  | AsnC family transcriptional regulator. | | pH 7.5 |
| 113 | RL4404 | *gelA* | Gel forming EPS production protein GelA. Regulated by RosR [84]. Mutation has no effect on 2-9 d biofilm formation or attachment to root hairs (90 min) at pH 6.5 or 7.5 [4]. Appears to have role in primary bulk root attachment at pH 6.5. | | pH 6.5 |
| 114 | RL4497 |  | Putative transmembrane protein, coiled-coil domain. | | pH 7.5 |
| 115 | RL4704 |  | Putative glyoxylase family protein, member of the VOC superfamily. Members of this family are known to detoxify methylglyoxal, formed as a by-product of lipid metabolism [85]. | | pH 7.0 |
|  |  |  |  | |  |
| ^1^ mutant made in this gene to assess attachment with Lux assay (5 genes)  ^2^ gene with less than 6 TA sites therefore INSeq classification should be treated with caution (20 genes) | | | |  |  |
|  | Transcriptional regulator (7 genes) | | | |  |
|  | RpoH1 binding site (CTTGAA-N15-16-CCTATAT) upstream of gene (5 genes) | | | |  |
|  | RpoH1/H2 binding site (CTTGAA-N15-16-CCTATCT) upstream of gene (2 genes) | | | |  |

1. Johnston AWB, Beringer JE. Identification of the *Rhizobium* strains in pea root nodules using genetic markers. J Gen Microbiol. 1975;87:343-50.

2. Perry BJ, Yost CK. Construction of a mariner-based transposon vector for use in insertion sequence mutagenesis in the *Rhizobiaceae*. BMC Microbiol. 2014;14:1-11.

3. Frederix M, Edwards A, McAnulla C, Downie JA. Co-ordination of quorum-sensing regulation in *Rhizobium leguminosarum* by induction of an anti-repressor. Mol Microbiol. 2011;81:994-1007.

4. Williams A, Wilkinson A, Krehenbrink M, Russo DM, Zorreguieta A, Downie JA. Glucomannan-mediated attachment of *Rhizobium leguminosarum* to pea root hairs is required for competitive nodule infection. J Bacteriol. 2008;190:4706-15.

5. Frederix M, Edwards A, Swiderska A, Stanger A, Karunakaran R, Williams A, et al. Mutation of *praR* in *Rhizobium leguminosarum* enhances root biofilms, improving nodulation competitiveness by increased expression of attachment proteins. Mol Microbiol. 2014;93:464-78.

6. Prell J, Mulley G, Haufe F, White JP, Williams A, Karunakaran R, et al. The PTS^Ntr^ system globally regulates ATP-dependent transporters in *Rhizobium leguminosarum*. Mol Microbiol. 2012;84:117-29.

7. Hood G. Physiological response of *Rhizobium leguminosarum* during bacteroid development. PhD Thesis, University of East Anglia. 2013.

8. Karunakaran R, Ramachandran VK, Seaman JC, East AK, Moushine B, Mauchline TH, et al. Transcriptomic analysis of *Rhizobium leguminosarum* b.v. *viciae* in symbiosis with host plants *Pisum sativum* and *Vicia cracca*. J Bacteriol. 2009;191:4002-14.

9. Kuznetsova E, Nocek B, Brown G, Makarova KS, Flick R, Wolf YI, et al. Functional diversity of haloacid dehalogenase superfamily phosphatases from *Saccharomyces cerevisiae:* BIOCHEMICAL, STRUCTURAL, AND EVOLUTIONARY INSIGHTS. J Biol Chem. 2015;290:18678-98.

10. Li J, Wang N. Genome-wide mutagenesis of *Xanthomonas axonopodis* pv. *citri* reveals novel genetic determinants and regulation mechanisms of biofilm formation. PLoS One. 2011;6:e21804.

11. Tsai TI, Li ST, Liu CP, Chen KY, Shivatare SS, Lin CW, et al. An Effective Bacterial Fucosidase for Glycoprotein Remodeling. ACS Chem Biol. 2017;12:63-72.

12. Gharzouli R, Carpéné MA, Couderc F, Benguedouar A, Poinsot V. Relevance of fucose-rich extracellular polysaccharides produced by *Rhizobium sullae* strains nodulating *Hedysarum coronarium* l. legumes. Appl Environ Microbiol. 2013;79:1764-76.

13. Bahat-Samet E, Castro-Sowinski S, Okon Y. Arabinose content of extracellular polysaccharide plays a role in cell aggregation of *Azospirillum brasilense*. FEMS Microbiol Lett. 2004;237:195-203.

14. Mintz KP, Fives-Taylor PM. *impA,* a gene coding for an inner membrane protein, influences colonial morphology of *Actinobacillus actinomycetemcomitans*. Infect Immun. 2000;68:6580-6.

15. Sahu SK, Zheng P, Yao N. Niclosamide Blocks Rice Leaf Blight by Inhibiting Biofilm Formation of *Xanthomonas oryzae*. Front Plant Sci. 2018;9:408.

16. Dozot M, Poncet S, Nicolas C, Copin R, Bouraoui H, Mazé A, et al. Functional characterization of the incomplete phosphotransferase system (PTS) of the intracellular pathogen *Brucella melitensis*. PLoS One. 2010;5.

17. Pfluger-Grau K, Gorke B. Regulatory roles of the bacterial nitrogen-related phosphotransferase system. Trends Microbiol. 2010;18:205-14.

18. Untiet V, Karunakaran R, Kramer M, Poole P, Priefer U, Prell J. ABC Transport Is Inactivated by the PTSNtr under Potassium Limitation in *Rhizobium leguminosarum* 3841. PLoS One. 2013;8.

19. Hoffmann JH, Linke K, Graf PC, Lilie H, Jakob U. Identification of a redox-regulated chaperone network. EMBO J. 2004;23:160-8.

20. Kumar CM, Mande SC, Mahajan G. Multiple chaperonins in bacteria--novel functions and non-canonical behaviors. Cell Stress Chaper. 2015;20:555-74.

21. Monteiro C, Fang X, Ahmad I, Gomelsky M, Römling U. Regulation of biofilm components in *Salmonella enterica* serovar Typhimurium by lytic transglycosylases involved in cell wall turnover. J Bacteriol. 2011;193:6443-51.

22. Niba ET, Naka Y, Nagase M, Mori H, Kitakawa M. A genome-wide approach to identify the genes involved in biofilm formation in *E. coli*. DNA Res. 2007;14:237-46.

23. Crépin S, Ottosen EN, Peters K, Smith SN, Himpsl SD, Vollmer W, et al. The lytic transglycosylase MltB connects membrane homeostasis and in vivo fitness of *Acinetobacter baumannii*. Mol Microbiol. 2018;109:745-62.

24. Scheurwater E, Reid CW, Clarke AJ. Lytic transglycosylases: bacterial space-making autolysins. Int J Biochem Cell Biol. 2008;40:586-91.

25. Neudorf KD, Yost CK. An uncharacterized gene coding a conserved lytic transglycosylase domain (RL4716) is required for proper cell envelope function in *Rhizobium leguminosarum*. FEMS Microbiol Lett. 2017;364.

26. Anderson BE, Baumstark BR, Bellini WJ. Expression of the gene encoding the 17-kilodalton antigen from *Rickettsia rickettsii*: transcription and posttranslational modification. J Bacteriol. 1988;170:4493-500.

27. Hamada M, Toyofuku M, Miyano T, Nomura N. cbb3-type cytochrome c oxidases, aerobic respiratory enzymes, impact the anaerobic life of *Pseudomonas aeruginosa* PAO1. J Bacteriol. 2014;196:3881-9.

28. Darwin AJ. Stress relief during host infection: The phage shock protein response supports bacterial virulence in various ways. PLoS Path. 2013;9:e1003388.

29. Wang Q, Frye JG, McClelland M, Harshey RM. Gene expression patterns during swarming in *Salmonella typhimurium*: genes specific to surface growth and putative new motility and pathogenicity genes. Mol Microbiol. 2004;52:169-87.

30. Yeats C, Rawlings ND, Bateman A. The PepSY domain: a regulator of peptidase activity in the microbial environment? Trends Biochem Sci. 2004;29:169-72.

31. Iijima S, Washio K, Okahara R, Morikawa M. Biofilm formation and proteolytic activities of *Pseudoalteromonas* bacteria that were isolated from fish farm sediments. Microbiol Biotechnol. 2009;2:361-9.

32. Minder AC, de Rudder KEE, Narberhaus F, Fischer HM, Hennecke H, Geiger O. Phosphatidylcholine levels in *Bradyrhizobium japonicum* membranes are critical for an efficient symbiosis with the soybean host plant. Mol Microbiol. 2001;39:1186-98.

33. Zhang Y, Cottet SE, Ealick SE. Structure of *Escherichia coli* AMP nucleosidase reveals similarity to nucleoside phosphorylases. Structure. 2004;12:1383-94.

34. Anantharaman V, Aravind L. The NYN domains: novel predicted RNAses with a PIN domain-like fold. Rna Biol. 2006;3:18-27.

35. Mathew R, Mukherjee R, Balachandar R, Chatterji D. Deletion of the *rpoZ* gene, encoding the omega subunit of RNA polymerase, results in pleiotropic surface-related phenotypes in *Mycobacterium smegmatis*. Microbiol. 2006;152:1741-50.

36. Bhardwaj N, Syal K, Chatterji D. The role of ω-subunit of *Escherichia coli* RNA polymerase in stress response. Genes Cells. 2018;23:357-69.

37. Weiss A, Moore BD, Tremblay MHJ, Chaput D, Kremer A, Shaw LN. The ω Subunit Governs RNA Polymerase Stability and Transcriptional Specificity in *Staphylococcus aureus*. J Bacteriol. 2017;199.

38. Hu C, Xiong N, Zhang Y, Rayner S, Chen S. Functional characterization of lipase in the pathogenesis of *Staphylococcus aureus*. Biochem Biophys Res Commun. 2012;419:617-20.

39. Kobayashi K. Gradual activation of the response regulator DegU controls serial expression of genes for flagellum formation and biofilm formation in *Bacillus subtilis*. Mol Microbiol. 2007;66:395-409.

40. Yoshida K, Takemoto Y, Sotsuka T, Tanaka K, Takenaka S. PhaP phasins play a principal role in poly-β-hydroxybutyrate accumulation in free-living *Bradyrhizobium japonicum*. BMC Microbiol. 2013;13:290.

41. Fernández L, Breidenstein EB, Song D, Hancock RE. Role of intracellular proteases in the antibiotic resistance, motility, and biofilm formation of *Pseudomonas aeruginosa*. Antimicrob Agents Chemother. 2012;56:1128-32.

42. Torres-Quesada O, Oruezabal RI, Peregrina A, Jofre E, Lloret J, Rivilla R, et al. The *Sinorhizobium meliloti* RNA chaperone Hfq influences central carbon metabolism and the symbiotic interaction with alfalfa. BMC Microbiol. 2010;10:-.

43. Dey S, Biswas C, Sengupta J. The universally conserved GTPase HflX is an RNA helicase that restores heat-damaged *Escherichia coli* ribosomes. J Cell Biol. 2018;217:2519-29.

44. Missiakas D, Schwager F, Raina S. Identification and characterization of a new disulfide isomerase-like protein (DsbD) in *Escherichia coli*. EMBO J. 1995;14:3415-24.

45. Valentini M, Filloux A. Biofilms and Cyclic di-GMP (c-di-GMP) Signaling: Lessons from *Pseudomonas aeruginosa* and Other Bacteria. J Biol Chem. 2016;291:12547-55.

46. Plate L, Marletta MA. Nitric oxide modulates bacterial biofilm formation through a multicomponent cyclic-di-GMP signaling network. Mol Cell. 2012;46:449-60.

47. Davies C, White SW, Nicholas RA. Crystal structure of a deacylation-defective mutant of penicillin-binding protein 5 at 2.3-A resolution. J Biol Chem. 2001;276:616-23.

48. van Heijenoort J. Formation of the glycan chains in the synthesis of bacterial peptidoglycan. Glycobiol. 2001;11:25r-36r.

49. Becker P, Hufnagle W, Peters G, Herrmann M. Detection of differential gene expression in biofilm-forming versus planktonic populations of *Staphylococcus aureus* using micro-representational-difference analysis. Appl Environ Microbiol. 2001;67:2958-65.

50. Pancholi V, Fischetti VA. alpha-enolase, a novel strong plasmin(ogen) binding protein on the surface of pathogenic streptococci. J Biol Chem. 1998;273:14503-15.

51. Modun B, Williams P. The staphylococcal transferrin-binding protein is a cell wall glyceraldehyde-3-phosphate dehydrogenase. Infect Immun. 1999;67:1086-92.

52. Bao S, Chen D, Yu S, Chen H, Tan L, Hu M, et al. Characterization of triosephosphate isomerase from *Mycoplasma gallisepticum*. FEMS Microbiol Lett. 2015;362:fnv140.

53. Uehara T, Suefuji K, Valbuena N, Meehan B, Donegan M, Park JT. Recycling of the anhydro-N-acetylmuramic acid derived from cell wall murein involves a two-step conversion to N-acetylglucosamine-phosphate. J Bacteriol. 2005;187:3643-9.

54. Uehara T, Suefuji K, Jaeger T, Mayer C, Park JT. MurQ Etherase is required by *Escherichia coli* in order to metabolize anhydro-N-acetylmuramic acid obtained either from the environment or from its own cell wall. J Bacteriol. 2006;188:1660-2.

55. Modzelan M, Kujawa M, Głąbski K, Jagura-Burdzy G, Kraszewska E. NudC Nudix hydrolase from *Pseudomonas syringae*, but not its counterpart from *Pseudomonas aeruginosa,* is a novel regulator of intracellular redox balance required for growth, motility and biofilm formation. Mol Microbiol. 2014;93:867-82.

56. Cox MM. The bacterial RecA protein as a motor protein. Ann Rev Microbiol. 2003;57:551-77.

57. Kumar KK, Srivastava R, Sinha VB, Michalski J, Kaper JB, Srivastava BS. *recA* mutations reduce adherence and colonization by classical and El Tor strains of *Vibrio cholerae*. Microbiol 1994;140 ( Pt 5):1217-22.

58. Schulte JE, Goulian M. The Phosphohistidine Phosphatase SixA Targets a Phosphotransferase System. Mbio. 2018;9:e01666-18.

59. Beloin C, Valle J, Latour-Lambert P, Faure P, Kzreminski M, Balestrino D, et al. Global impact of mature biofilm lifestyle on *Escherichia coli* K-12 gene expression. 2004;51:659-74.

60. Niba ET, Naka Y, Nagase M, Mori H, Kitakawa M. A genome-wide approach to identify the genes involved in biofilm formation in E. coli. 2007;14:237-46.

61. Wahl A, My L, Dumoulin R, Sturgis JN, Bouveret E. Antagonistic regulation of *dgkA* and *plsB* genes of phospholipid synthesis by multiple stress responses in *Escherichia coli*. Mol Microbiol. 2011;80:1260-75.

62. Jerga A, Lu YJ, Schujman GE, de Mendoza D, Rock CO. Identification of a soluble diacylglycerol kinase required for lipoteichoic acid production in *Bacillus subtilis*. J Biol Chem. 2007;282:21738-45.

63. Yepes A, Schneider J, Mielich B, Koch G, García-Betancur JC, Ramamurthi KS, et al. The biofilm formation defect of a *Bacillus subtilis* flotillin-defective mutant involves the protease FtsH. Mol Microbiol. 2012;86:457-71.

64. Skagia A, Zografou C, Vezyri E, Venieraki A, Katinakis P, Dimou M. Cyclophilin PpiB is involved in motility and biofilm formation via its functional association with certain proteins. Genes Cells. 2016;21:833-51.

65. Schumann W. FtsH--a single-chain charonin? FEMS Microbiol Rev. 1999;23:1-11.

66. Kihara A, Akiyama Y, Ito K. A protease complex in the *Escherichia coli* plasma membrane: HflKC (HflA) forms a complex with FtsH (HflB), regulating its proteolytic activity against SecY. EMBO J. 1996;15:6122-31.

67. Hinderhofer M, Walker CA, Friemel A, Stuermer CA, Möller HM, Reuter A. Evolution of prokaryotic SPFH proteins. BMC Evol Biol. 2009;9:10.

68. Duan E, Wang Y, Liu L, Zhu J, Zhong M, Zhang H, et al. Pyrophosphate: fructose-6-phosphate 1-phosphotransferase (PFP) regulates carbon metabolism during grain filling in rice. Plant Cell Rep. 2016;35:1321-31.

69. Janczarek M, Skorupska A. Regulation of *pssA* and *pssB* gene expression in *Rhizobium leguminosarum* bv. *trifolii* in response to environmental factors. Antonie Van Leeuwenhoek. 2004;85:217-27.

70. Ivashina TV, Khmelnitsky MI, Shlyapnikov MG, Kanapin AA, Ksenzenko VN. The *pss4* gene from *Rhizobium leguminosarum* bv *viciae* VF39 - cloning, sequence and the possible role in polysaccharide production and nodule formation. Gene. 1994;150:111-6.

71. Oke V, Rushing BG, Fisher EJ, Moghadam-Tabrizi M, Long SR. Identification of the heat-shock sigma factor RpoH and a second RpoH-like protein in *Sinorhizobium meliloti*. Microbiol. 2001;147:2399-408.

72. Makarova KS, Aravind L, Galperin MY, Grishin NV, Tatusov RL, Wolf YI, et al. Comparative genomics of the Archaea (Euryarchaeota): evolution of conserved protein families, the stable core, and the variable shell. Genome Res. 1999;9:608-28.

73. Clissold PM, Ponting CP. PIN domains in nonsense-mediated mRNA decay and RNAi. Current biology : CB. 2000;10:R888-90.

74. Domínguez-Ferreras A, Pérez-Arnedo R, Becker A, Olivares J, Soto MJ, Sanjuán J. Transcriptome profiling reveals the importance of plasmid pSymB for osmoadaptation of *Sinorhizobium meliloti*. J Bacteriol. 2006;188:7617-25.

75. Pittman JR, Buntyn JO, Posadas G, Nanduri B, Pendarvis K, Donaldson JR. Proteomic analysis of cross protection provided between cold and osmotic stress in *Listeria monocytogenes*. J Proteome Res. 2014;13:1896-904.

76. Vedam V, Haynes JG, Kannenberg EL, Carlson RW, Sherrier DJ. A *Rhizobium leguminosarum* lipopolysaccharide lipid-A mutant induces nitrogen-fixing nodules with delayed and defective bacteroid formation. Mol Plant Microbe Interact. 2004;17:283-91.

77. Vanderlinde EM, Muszyński A, Harrison JJ, Koval SF, Foreman DL, Ceri H, et al. *Rhizobium leguminosarum* biovar *viciae* 3841, deficient in 27-hydroxyoctacosanoate-modified lipopolysaccharide, is impaired in desiccation tolerance, biofilm formation and motility. Microbiol. 2009;155:3055-69.

78. Doerrler WT, Sikdar R, Kumar S, Boughner LA. New functions for the ancient DedA membrane protein family. J Bacteriol. 2013;195:3-11.

79. Rodionov DA, Vitreschak AG, Mironov AA, Gelfand MS. Comparative genomics of the vitamin B12 metabolism and regulation in prokaryotes. J Biol Chem. 2003;278:41148-59.

80. Campbell GRO, Taga ME, Mistry K, Lloret J, Anderson PJ, Roth JR, et al. *Sinorhizobium meliloti bluB* is necessary for production of 5,6-dimethylbenzimidazole, the lower ligand of B12. Proc Natl Acad Sci U S A. 2006;103:4634-9.

81. Taga ME, Walker GC. *Sinorhizobium meliloti* Requires a Cobalamin-Dependent Ribonucleotide Reductase for Symbiosis With Its Plant Host. Mol Plant Microbe Interact. 2010;23:1643-54.

82. Leisico F, D VV, Figueiredo TA, Silva M, Cabrita EJ, Sobral RG, et al. First insights of peptidoglycan amidation in Gram-positive bacteria - the high-resolution crystal structure of Staphylococcus aureus glutamine amidotransferase GatD. Sci Rep. 2018;8:5313.

83. Pedersen LB, Murray T, Popham DL, Setlow P. Characterization of *dacC*, which encodes a new low-molecular-weight penicillin-binding protein in *Bacillus subtilis*. J Bacteriol. 1998;180:4967-73.

84. Rachwał K, Matczyńska E, Janczarek M. Transcriptome profiling of a *Rhizobium leguminosarum* bv. *trifolii rosR* mutant reveals the role of the transcriptional regulator RosR in motility, synthesis of cell-surface components, and other cellular processes. BMC Genom. 2015;16:1111.

85. Singla-Pareek SL, Reddy MK, Sopory SK. Genetic engineering of the glyoxalase pathway in tobacco leads to enhanced salinity tolerance. Proc Natl Acad Sci U S A. 2003;100:14672-7.
